# Liver Metastasis Modulate Responses of Suppressive Macrophages and Exhausted T Cells to Immunotherapy Revealed by Single Cell Sequencing

**DOI:** 10.1002/ggn2.202200002

**Published:** 2022-10-11

**Authors:** Qiming Zhang, Siyuan Liu, Yedan Liu, Dev Bhatt, Juan Estrada, Brian Belmontes, Xianwen Ren, Jude Canon, Wenjun Ouyang

**Affiliations:** ^1^ Beijing Advanced Innovation Center for Genomics (ICG) School of Life Sciences Peking University Beijing 100871 China; ^2^ Biomedical Pioneering Innovation Center Ministry of Education Key Laboratory of Cell Proliferation and Differentiation Beijing 100871 China; ^3^ Department of Inflammation and Oncology Amgen Research, Amgen, Inc. South San Francisco CA 94080 USA; ^4^ Present address: Department of Biology The David H. Koch Institute for Integrative Cancer Research at MIT Massachusetts Institute of Technology Cambridge MA 02139 USA; ^5^ Present address: Oncovalent Therapeutics Thousand Oaks CA 91320 USA; ^6^ Present address: Department of Inflammation and Fibrosis, Research, Gilead Science CA 94404 USA

**Keywords:** anti‐PD‐1 treatment, colorectal cancer liver metastases, exhausted CD8^+^ T cells, immunosuppressive tumor‐associated macrophages, immunotherapy resistance, interferon signatures, single cell RNA‐sequencing

## Abstract

Liver metastasis is associated with immunotherapy resistance, although the underlying mechanisms remain incompletely understood. By applying single cell RNA‐sequencing to a concurrent subcutaneous and liver tumor murine model to recapitulate liver metastases, it is identified that subsets within tumor‐infiltrating exhausted CD8^+^ T (Tex) cells and immunosuppressive tumor‐associated macrophages (TAMs) display opposite responses to concurrent liver tumors and anti‐PD‐1 treatment, suggesting a complex immune regulating network. Both angiogenic and interferon‐reactive TAMs show increased frequencies in implanted liver tumors, and anti‐PD‐1 treatment further elevates the frequencies of angiogenic TAMs. Such TAMs frequencies negatively correlate with the proportions of cytotoxic T cell subsets. Further, expression of interferon‐stimulated genes in TAMs is dramatically reduced under effective anti‐PD‐1 treatment, while such tendencies are diminished in mice with implanted liver tumors. Therefore, the study indicates that liver metastases could increase immunosuppressive TAMs frequencies and inhibit Tex responses to PD‐1 blockade, resulting in compromised systemic antitumor immunity and limited immunotherapy efficacy.

## Graphical abstract

By applying single cell RNA‐sequencing to a concurrent subcutaneous and liver tumor murine model to recapitulate liver metastases, tumor‐infiltrating exhausted CD8^+^ T (Tex) cells, immunosuppressive tumor‐associated macrophages, and interferon‐stimulated gene scores are identified to display opposite responses to liver metastasis and anti‐PD‐1 treatment, suggesting a complex immune regulating network.

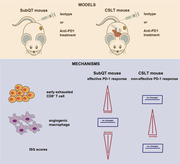

## 1 Introduction

Immune‐checkpoint blockade (ICB) can regulate the inhibitory signaling pathways in T cells and maintain antitumor responses in patients with a variety of cancers, leading to significant clinical benefits.^[^ ^1^, ^2^ ^]^ ICB responses have been reported to be associated with multiple factors, including mutation burden,^[^ ^3^ ^]^ the infiltration of cytotoxic CD8^+^ T cells,^[^ ^4^ ^]^ and the presence of tertiary lymphoid structures.^[^ ^5^, ^6^ ^]^ Patients with liver metastases often exhibit reduced response to anti‐PD‐1 and shortened progression‐free survival compared with non‐metastatic patients in different cancer types.^[^ ^7^, ^8^ ^]^ Liver metastasis has been reported to potentially remodel the tumor microenvironment (TME) including macrophages, regulatory T cells (Tregs), and NK cells, and subsequently restrain immunotherapy.^[^ ^9^, ^10^, ^11^, ^12^ ^]^ However, it remains unclear how liver metastasis would influence the TME and modulate systemic antitumor immunity through enhancement of Treg activation and depletion of T effector cells.

Single‐cell RNA sequencing (scRNA‐seq) has become a powerful tool for dissecting the complexity of TME, revealing the cell diversity and functional states in unprecedented details.^[^ ^13^ ^]^ Based on scRNA‐seq, the heterogeneity of tumor‐infiltrating immune cells in clinical samples from different cancer types has been revealed.^[^ ^14^, ^15^, ^16^, ^17^, ^18^, ^19^, ^20^, ^21^, ^22^ ^]^ Previously, we have characterized multiple immune cell populations that might be critical for responses to ICB, including exhausted T (Tex) cells,^[^ ^19^, ^20^, ^21^ ^]^ Th1‐like cells,^[^ ^17^ ^]^ *SPP1* ^+^ tumor‐associated macrophages (TAMs),^[^ ^14^ ^]^ and *LAMP3* ^+^ dendritic cells (DCs).^[^ ^15^ ^]^ Additional scRNA‐seq studies have provided further insights into the underlying mechanisms and immune‐based biomarkers associated with responses to ICB.^[^ ^23^, ^24^ ^]^ However, the underlying mechanisms and deterministic factors of responses to ICB in clinical samples remain incompletely understood due to obstacles in obtaining patient samples and carrying out functional perturbations. Recent scRNA‐seq studies have been simultaneously applied on murine models and clinical samples to perform comparative studies of various cell populations in tumors, and further explored the effects of manipulating those cell populations conserved across mice and human.^[^ ^14^, ^25^, ^26^, ^27^, ^28^ ^]^ We thus reasoned that single‐cell analyses of murine models reflecting the liver metastasis process can facilitate the study of systemic antitumor immunity to better understand the impacts of metastases to ICB responses.

Here, we established a concurrent subcutaneous (SubQ) and liver tumor (CSLT) murine model and recapitulated reduced responses to anti‐PD‐1 treatment compared with SubQ‐only mice. With scRNA‐seq data on CD45^+^ immune cell populations of these mice, we compared our data with colorectal cancer (CRC) patients and revealed that most tumor‐infiltrating immune cell subsets were conserved with similar gene expression patterns.^[^ ^14^ ^]^ The pivotal elements in TME including terminally Tex cells and immunosuppressive TAMs could reflect their equivalent functions in humans, providing insights into how liver metastases would possibly diminish responses to immunotherapy.

## 2 Results

### 2.1 Diminished Anti‐PD‐1 Response in the CSLT Murine Model

**Figure 1:**
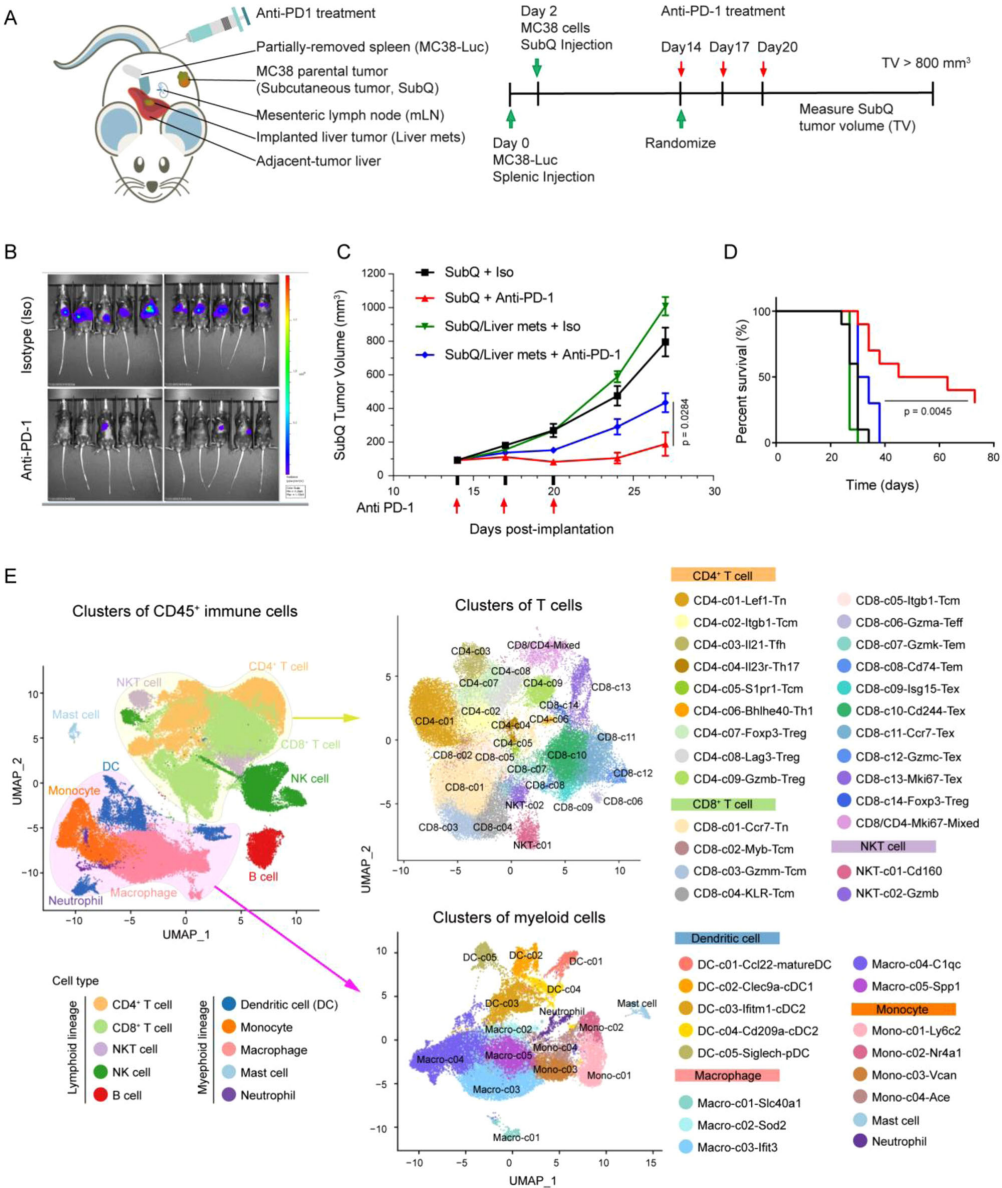
Immune cell types in the concurrently subcutaneous and liver tumor (CSLT) murine models. A) Schematic for the CSLT murine model using the MC38 tumor cell line. B) Luciferase monitoring the growth of implanted liver tumors. Upper items showed the isotype groups, and bottom items showed the anti‐PD‐1 groups. C) Growth curves of SubQ tumors in different experimental groups including implanted liver tumors and anti‐PD‐1 treatment. D) Survival curves of mice in different experimental groups including implanted liver tumors and anti‐PD‐1 treatment. E) combined UMAP plots showing the major immune cell populations (left) and clusters of T (top right) and myeloid cells (lower right) in CSLT and SubQ‐only mice based on scRNA‐seq. Dot represents individual cells, and colors represent different major cell types or different clusters.

To evaluate the impact of liver metastasis on systemic immune reactions and responses to anti‐PD‐1 treatment, we developed a mouse model based on a two‐site tumor system with concurrent implantation of SubQ and liver tumor to mimic the liver metastasis as previously described.^[^ ^29^ ^]^ Specifically, we established liver metastases by injecting MC38 tumor cells to partially‐removed spleens, and SubQ tumors were injected 2 days after the splenic inoculation (**Figure** **1**A). The MC38 cells injected in spleens were engineered to express luciferase to monitor the growth of metastatic tumors (Figure 1B). As control, SubQ‐only mice were also established without implanting MC38 tumors in livers. Both SubQ and liver tumors were randomized based on their size to comparable groups before treatments. In SubQ‐only mice, tumors responded to anti‐PD‐1 treatment as expected,^[^ ^10^, ^11^ ^]^ evidenced by significantly reduced tumor growth and enhanced survival rates (Figure 1C,D, and Figure S1D, Supporting Information). The surgical procedure of spleen partial‐removal for establishing liver metastasis did not influence effective responses of SubQ tumors to PD‐1 blockade (Figure S1F–H, Supporting Information). However, the presence of liver tumors significantly limited the therapeutic benefits of anti‐PD‐1 treatment on SubQ tumors (Figure 1C,D, and Figure S1D, Supporting Information), despite the fact that the liver implanted MC38 tumors themselves showed responses to anti‐PD‐1 (Figure S1A–C, Supporting Information). These data suggested that tumors growing in the liver hampered the efficacy of PD‐1 blockade to tumors developed in other organs (i.e. SubQ in our study), similar to the reduced immunotherapy efficacy in melanoma and NSCLC patients with liver metastasis.^[^ ^7^, ^8^ ^]^ Such observations supported that this model could be used to dissect the immune mechanisms of ICB resistance in liver metastasized CRC patients, while our models could only study the impact of the cooccurrence of liver tumor on the efficacy of PD‐1 blockade tumors in other organs but not *de novo* liver metastasis.

### 2.2 scRNA‐Seq Profiling Maps Immune Cell Populations in Mouse and Human

To interrogate the alterations of TME components in different conditions, we performed flow cytometry (FACS) analyses on tumor‐infiltrating immune populations of the aforementioned groups, but did not observe any significant changes (Figure S1E, Supporting Information). Therefore, we next performed MULTI‐seq on immune cells from mice with or without liver tumors, and generated 5′ scRNA‐seq and T cell receptor‐sequencing (TCR‐seq) libraries to study how liver metastases would impact the TME in detail (Figure S2A, Supporting Information).^[^ ^30^ ^]^ Specifically, CD45^+^ total leukocytes were collected from SubQ, liver tumors, and tumor‐adjacent liver tissues. We also collected CD19^–^TCR^–^ leukocytes from spleen and enriched memory T cells (TCRβ^+^CD44^hi^) from mesenteric lymph nodes (mLN). Individual samples from each mouse were labeled with lipid‐tagged indices and mixed before scRNA‐seq as previously described.^[^ ^30^ ^]^ We obtained 91 902 single‐cell transcriptomes after filtering low‐quality data, of which 33 623 immune cells could be traced by lipid‐tagged indices to specific samples. Among all the cells, 29 479 T cells had one pair of full‐length productive α and β chains, and were used to trace TCR clonal expansion.

To define the major and minor cell populations in our models, we performed graph‐based clustering on all the 91 902 single‐cell transcriptome data and identified expected immune populations of both myeloid (monocytes, macrophages, DCs, mast cells, and neutrophils) and lymphoid lineages (T cells, NK cells, and B cells) (Figure 1E and Figure S2B, Supporting Information).^[^ ^31^ ^]^ In accordance with previous observations that certain cell populations in humans and mice were conserved,^[^ ^14^, ^25^ ^]^ both major immune cell types and their subsets in our data highly resembled those in CRC patients (Figure S2C–G, Supporting Information).^[^ ^14^ ^]^ In total, we identified 50 cell clusters, 16 for myeloid lineages and 34 for lymphoid lineages (Table S1, Supporting Information). These clusters were mixed well in different treatment groups and individual mice (Figure S3A,B, Supporting Information), suggesting minimum batch effects. Many of these clusters exhibited distinct tissue preferences. For lymphocytes, B cell clusters were present mainly in adjacent livers and mLNs (Figure S4C–E and Table S2, Supporting Information). Spleen‐resident (NK‐c01‐S1pr5), liver‐resident (NK‐c02‐Cd160), and tumor‐enriched NK cells (NK‐c03‐Ccl5 ≈ NK‐c06‐Stmn1) were also identified (Figure S4A,B,E, Supporting Information), similar to the tissue‐enriched NK cell population in CRC patients (Figure S4G, Supporting Information).^[^ ^14^ ^]^ Tumor‐enriched NK cells contained four subpopulations that highly expressed genes related to cytotoxicity (NK‐c03‐Ccl5 and NK‐c05‐Gzmc), interferon response (NK‐c04‐Isg15), and proliferation (NK‐c06‐Stmn1) (Figure S4F and Table S3, Supporting Information), indicative of distinct phenotypes of NK cells in tumors. In addition to canonical CD8^+^ T and CD4^+^ T cell clusters ‐ naive (Tn), central memory (Tcm), effector memory (Tem), effector (Teff), CD4^+^ helper (Th), and Treg cells, we also identified CD8^+^ Tex clusters and two NKT cell populations (Figure 1E; Figure S5A–D and Table S4, Supporting Information), most of which were consistent with previous studies.^[^ ^17^, ^32^ ^]^ Tex and Treg populations accounted for 87.5% and 68.6% of CD8^+^ and CD4^+^ T cells in tumors, respectively (Figure S5E,F, Supporting Information), consistent with previous observations that Tex and Treg populations were enriched in tumors of different cancer types.^[^ ^19^, ^21^ ^]^

Within the myeloid lineages, 4 monocyte clusters, 5 macrophage clusters, and 5 DC clusters were identified (Figure 1E; Figure S6A,B and Table S5, Supporting Information). For DC populations, we obtained plasmacytoid DC (pDC, DC‐c05‐Siglech), cDC1 (DC‐c02‐Clec9a), cDC2 (DC‐c03‐Ifit3 and DC‐04‐Cd209a) and the recently described “activated” and “regulatory” DCs (DC‐c01‐Ccl22) (Figure S6A, Supporting Information).^[^ ^14^, ^15^, ^25^, ^33^ ^]^ cDC1s and pDCs exhibited preferential enrichment in adjacent liver, while activated DCs were more enriched in liver tumors. The two cDC2 populations, DC‐c03‐Ifit3 and DC‐c04‐Cd209a, showed enrichment in tumors and non‐cancer tissues (spleen and adjacent liver), respectively (Figure S6D, Supporting Information). Compared to DC‐c04‐Cd209a, tumor‐infiltrating cDC2s (DC‐c03‐Ifit3) highly expressed activated (*Cd83*, *Cd74*, and *H2‐Ab1*), inflammatory (*Isg15*, *Ly6a*, *Cxcl16*, *Fos* and *Junb*) and inhibitory (*Il1b* and *Il1r2*) genes (Figure S6E, Supporting Information), suggesting that the phenotypes of cDC2s in tumors were heterogeneous and might be influenced by the TME. Of monocyte clusters, Mono‐c01‐Ly6c2 and Mono‐c02‐Nr4a1 showed preferential enrichment in spleens and adjacent livers. By comparison, Mono‐c03‐Vcan and Mono‐c04‐Ace were enriched in tumors (Figure S6D, Supporting Information) and similar to classical CD14^hi^CD16^–^ and CD14^+^CD16^hi^ monocytes in CRC patients, respectively (Figure S6C, Supporting Information).^[^ ^14^ ^]^ Within the 5 macrophage clusters, only Macro‐c01‐Slc40a1 showed non‐cancer tissue preference, whereas the remaining clusters were enriched in tumors and thus denoted as TAMs (Figure S6B,D, Supporting Information). While Macro‐c04‐C1qc and Macro‐c05‐Spp1 were equivalent to *C1QC* ^+^ and *SPP1* ^+^ TAMs in CRC patients,^[^ ^14^ ^]^ Macro‐c02‐Sod2 and Macro‐c03‐Ifit3 did not resemble specific populations in CRC patients (Figure S2E, Supporting Information). These data suggest that both conserved and divergent immune cell populations exist in CRC patients and CSLT mice.

### 2.3 Concurrent Liver Tumors Diminished Treg and NK Cells Responses to Anti‐PD‐1

**Figure 2:**
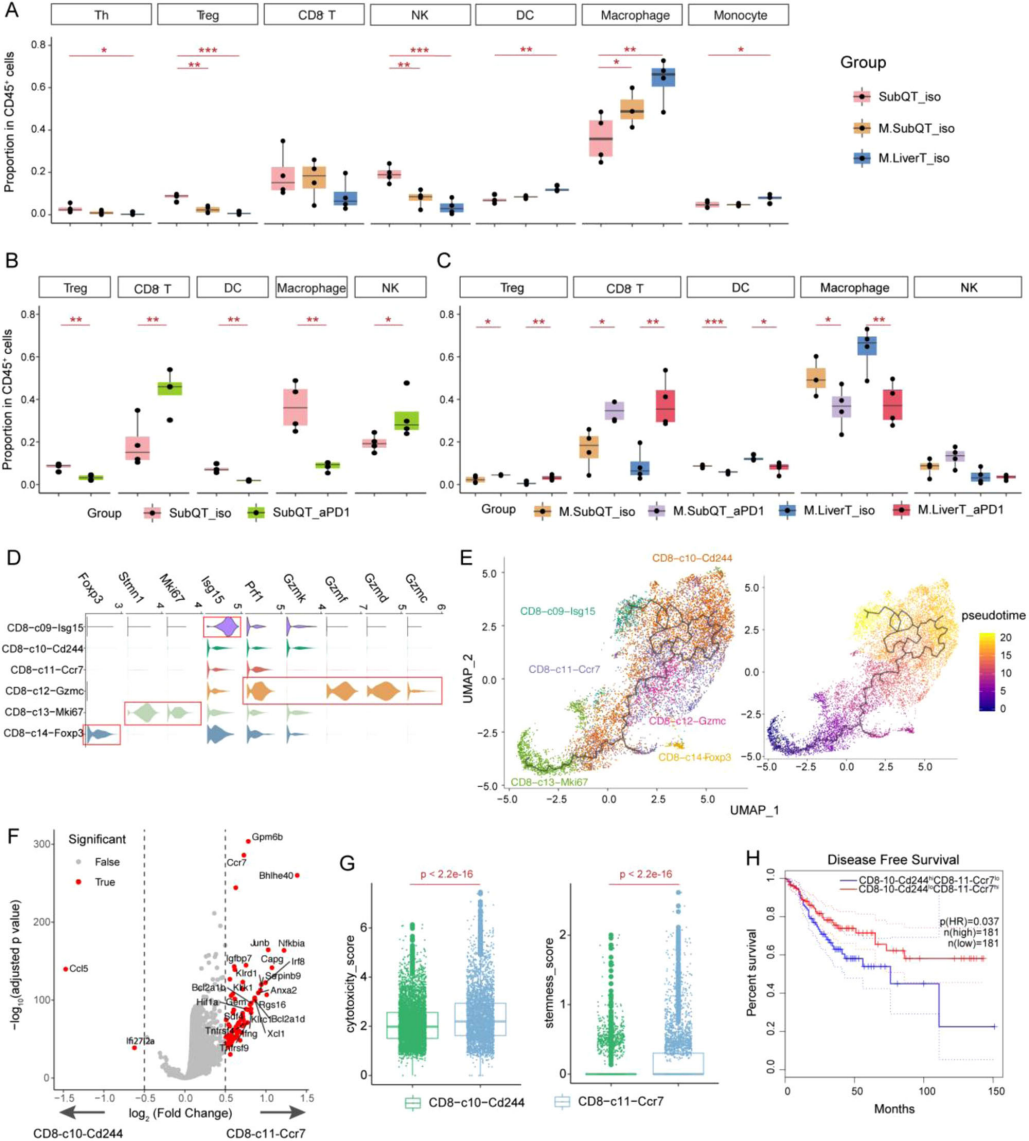
Alterations of major cell populations in different groups. A) Boxplots showing the frequency changes of the major cell populations in CSLT mice compared with SubQ‐only. *p < 0.05; **p < 0.01; ***p < 0.001. One‐sided *t*‐test. Boxplots showing the anti‐PD‐1‐induced frequency changes of the major cell populations in B) CSLT mice compared with C) SubQ‐only mice. *p < 0.05; **p < 0.01; ***p < 0.001. One‐sided *t*‐test. D) violin plot showing the expression of marker genes for Tex cell subsets. E) UMAP plot showing the trajectory relationship of Tex subsets based on Monocle3.^[^ ^35^ ^]^ Colors represent different clusters in the left panel, and represent pseudotime in the right panel. F) Volcano plot showing the differentially expressed genes between Cd8‐c10‐Cd244 and Cd8‐c11‐Ccr7. G) Box plots showing higher cytotoxicity and stemness scores of Cd8‐11‐Ccr7 than Cd8‐10‐Cd244. One‐sided t‐test. H) Kaplan‐Meier DFS curves of TCGA COAD and READ patients grouped by the gene signature expression of CD8‐10‐Cd244 and CD8‐11‐Ccr7. HR, hazard ratio. Multivariate Cox regression. SubQT: subcutaneous tumors in SubQ‐only mice; M.SubQT: subcutaneous tumors in CSLT mice; M.LiverT: liver tumors in CSLT mice; iso: isotype; aPD1: anti‐PD‐1 treatment.

To understand the mechanisms underlying the loss of efficacy to anti‐PD‐1 in SubQ when liver tumors were implanted, we investigated the alterations of major leukocyte populations under different conditions. In SubQ‐only mice, anti‐PD‐1 treatment significantly increased the percentages of NK and CD8^+^ T cells, and reduced the frequencies of macrophages, DCs, and Tregs (**Figure** **2**B), consistent with the previously reported effects of anti‐PD‐1 on various leukocyte subsets.^[^ ^34^ ^]^

Compared with SubQ‐only mice, liver tumors in CSLT mice contained significantly increased DCs and monocytes as well as decreased Th cells. Both SubQ and liver tumors in CSLT mice exhibited higher percentages of macrophages and lower fractions of Tregs and NK cells (Figure 2A), supporting that tumors growing in the liver not only affected leukocyte infiltration locally but also influenced the immune infiltration in distant tumor sites.^[^ ^10^, ^11^ ^]^ Anti‐PD‐1 increased the percentage of tumor‐infiltrating CD8^+^ T cells and decreased macrophages and DCs in both SubQ and liver tumors, similar to the alterations in SubQ‐only mice. However, anti‐PD‐1 did not alter the fractions of NK cells in CSLT mice (Figure 2C). In addition, instead of decreased Treg percentages in SubQ‐only mice (Figure 2B), anti‐PD‐1 enhanced Treg populations in both tumor sites of CSLT mice (Figure 2C), consistent with the previous notion that Tregs controlled the suppression of distant tumor immunity in the presence of liver tumors.^[^ ^10^ ^]^ Taken together, these data suggested that metastatic tumors growing in the liver could impact systemic responses of major immune populations toward PD‐1 blockade in vivo.

### 2.4 Liver Tumors Would Decrease Anti‐PD‐1 Responses of Early Exhausted CD8^+^ T Cells

We next distinguished the expression features of the six Tex subsets in our data. The two major Tex populations CD8‐c11‐Ccr7 and CD8‐c10‐Cd244 highly expressed canonical exhausted markers, including *Pdcd1*, *Tox*, *Lag3*, *Ctla4*, and *Tigit* (Figure S2F, Supporting Information). CD8‐c11‐Ccr7 had higher expression of *Ccr7*, *Il7r*, *Bhlhe40*, and *Ifng* compared to CD8‐c10‐Cd244 (Figure 2F), implying the early exhausted status of CD8‐c11‐Ccr7 and the terminally exhausted characteristics of CD8‐c10‐Cd244. Indeed, CD8‐c11‐Ccr7 exhibited higher stemness score (defined by the mean expression of *Ccr7*, *Il7r*, *Sell*, and *Tcf7*) and cytotoxicity score (defined by the mean expression of *Ifng*, *Nkg7*, *Prf1*, *Ccl3*, and *Ccl4*) than CD8‐c10‐Cd244 (Figure 2G), consistent with the previous study.^[^ ^36^ ^]^ Survival analyses on TCGA CRC patients also revealed that the ratio of early and terminally Tex cells was related to better prognosis (Figure 2H). CD8‐c13‐Mki67 highly expressed proliferation genes, similar to the Tex^porg2^ cells in LCMV mice (Figure 2D).^[^ ^37^ ^]^ CD8‐c14‐Foxp3 co‐expressed markers of T cell exhaustion like *Pdcd1* and marker genes of Tregs like *Foxp3*. Compared with CD4^+^ Tregs (CD4‐c09‐Gzmb), genes including *Cd8a*, *Nkg7*, *Klrc1*, and *Klrd1* were highly expressed in CD8‐c14‐Foxp3 (Figure S7B,C, Supporting Information), indicating that they might harbor both Treg and cytolytic characteristics, consistent with the previously reported CD8^+^ Tregs in human.^[^ ^17^, ^21^ ^]^ Trajectory analyses based on monocle3^[^ ^35^ ^]^ suggested that the developmental relationships among these Tex clusters in tumors might begin with proliferative Tex (CD8‐c13‐Mki67), followed by early exhausted states (CD8‐c11‐Ccr7 and CD8‐c12‐Gzmc) and ended with terminally exhausted states (CD8‐c09‐Isg15 and CD8‐c10‐Cd244), while regulatory state CD8‐c14‐Foxp3 cells appeared to form a side branch (Figure 2E and Figure S7A, Supporting Information), although this process needs further experimental validation.

**Figure 3:**
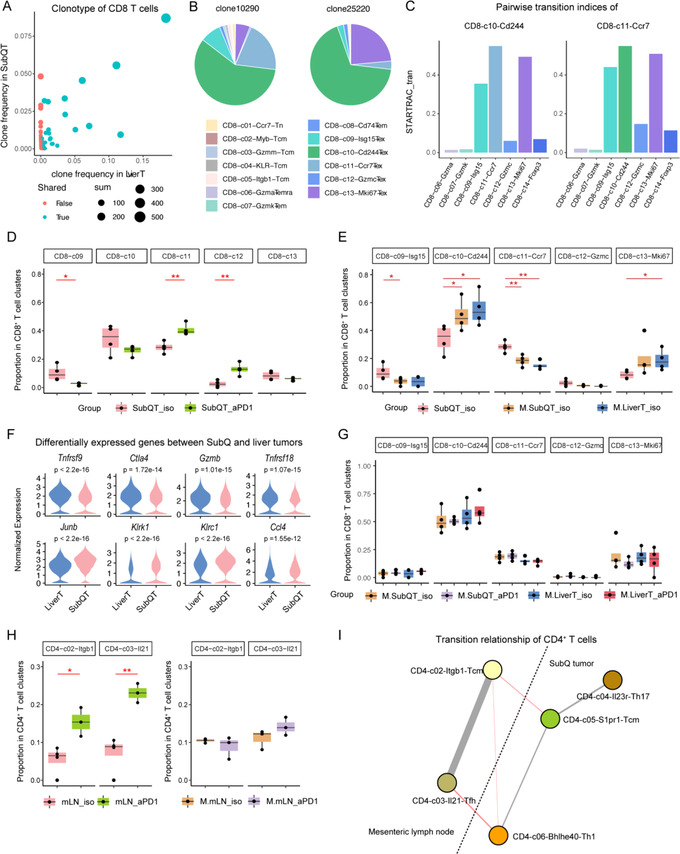
Alterations of T cell subsets in different conditions including implanted liver tumors and PD‐1 blockade. A) Scatter plot showing the shared clonotypes in CD8^+^ T cells between SubQ and liver tumors. Colors represent whether a clonotype is shared (blue) or not (red). Dot size represents cell numbers that a clonotype contains. B) Pie charts showing the fractions of different CD8^+^ T cell subsets in the two large clonotypes clone10290 and clone25220. C) Barplots showing the pairwise transition indices of CD8‐10‐Cd244 and CD8‐11‐Ccr7 with other T cell subsets. D) Boxplots showing that PD‐1 blockade induced changes in the abundance of CD8‐11‐Ccr7 and CD8‐12‐Gzmc, and the reduction of CD8‐09‐Isg15. *p < 0.05; **p < 0.01; ***p < 0.001. One‐sided *t*‐test. E) boxplots showing the increase of CD8‐10‐Cd244 and the decrease of CD8‐11‐Ccr7 in CSLT mice compared with SubQ‐only mice. *p < 0.05; **p < 0.01; ***p < 0.001. One‐sided *t*‐test. F) Violin plots showing several genes differentially expressed on CD8‐10‐Cd244 in the two tumor sites. One‐sided *t*‐test. G) Boxplots showing only moderate changes of Tex subsets induced by PD‐1 blockade in different tumor groups. *p < 0.05; **p < 0.01; ***p < 0.001. One‐sided *t*‐test. H) boxplots showing that PD‐1 blockade induced the abundance of CD4‐02‐Itgb1 and CD4‐03‐Il21 in mLN in SubQ‐only mice. *p < 0.05; **p < 0.01; ***p < 0.001. One‐sided *t*‐test. I) Node‐link plot showing the transitional relationship of mLN‐enriched and tumor‐enriched CD4^+^ T cell subsets. SubQT: subcutaneous tumors in SubQ‐only mice; M.SubQT: subcutaneous tumors in CSLT mice; M.LiverT: liver tumors in CSLT mice; mLN: mLN in SubQ‐only mice; M.mLN: mLN in CSLT mice; iso: isotype; aPD1: anti‐PD‐1 treatment.

The previous study showed that liver metastasis could induce systemic loss of antigen‐specific T cells,^[^ ^11^ ^]^ we thus also examined the clonotype variations of our T cell populations in different groups. Indeed, the presence of implanted liver tumors diminished the T cell diversity and clones in SubQ tumors for total CD8^+^, Tex, and Th cells but increased the diversity of Tregs. For specific clusters, intratumoral Tex and Th1 clusters also reduced their clonal diversity in mice with liver tumors, while activated Tregs showed accelerated clonal diversity (Table S6, Supporting Information). Such observations indicated that liver metastasis would induce clonal loss of tumor‐killing T cells but increase suppressive Tregs in SubQ tumors. In addition, we examined the T cell clone linkage between the two tumor sites. Based on STARTRAC, we observed that the shared TCR clones between SubQ and liver tumors were highly expanded (**Figure** **3**A and Figure S8B–D, Supporting Information). Zooming in on the top expanded clones, we observed that they were mainly composed of Tex cells (Figure 3B). We also attempt to compare the changes in TCR sharing in isotype and PD‐1 treatment groups. Although the highly shared TCR clonotype (more than 0.25 separated by the gray lines) numbers in isotype and PD‐1 treatment groups are similar, the clone size in the isotype group is much higher than the treatment group (Figure S8A, Supporting Information). These results indicate that although PD‐1 treatment is supposed to include more activated T cells, it is likely the opposite pattern in the metastasis circumstance, supporting metastasis associated ICB resistance. In addition, CD8‐c10‐Cd244 and CD8‐c11‐Ccr7 both showed higher pairwise STARTRAC‐tran indices connecting to the remaining Tex subsets compared with non‐Tex clusters (Figure 3C), further supporting the developmental connections among these Tex subsets. These data also indicated that clonal CD8^+^ T cells at both tumor sites might derive from common progenitors.

In both SubQ‐only and CSLT mice, anti‐PD‐1 consistently increased the total tumor infiltrating CD8^+^ T cells, including the infiltrates in SubQ and liver tumors (Figure 2B,C). We thus reasoned that the effects of liver tumors on CD8^+^ T cells might be distinct in different sub‐populations. Anti‐PD‐1 treatment in SubQ‐only mice caused an elevation of early Tex subsets including CD8‐c11‐Ccr7 and CD8‐c12‐Gzmc, but a reduced proportion of CD8‐c09‐Isg15 (Figure 3D). Since CD8‐c12‐Gzmc highly expressed a broader range of effector‐related genes including granzyme molecules (*Gzmc*, *Gzmd*, *Gzmf*) and *Prf1* compared to other Tex subsets (Figure 2D), the increase of this population indicated enhanced cytotoxicity of CD8^+^ T cells in SubQ after anti‐PD‐1 treatment. By contrast, in CSLT mice, concurrent liver tumors significantly increased CD8‐c10‐Cd244 but decreased CD8‐c11‐Ccr7 frequencies in both SubQ and liver tumors (Figure 3E). In addition, compared with SubQ tumors, the CD8‐c10‐Cd244 subset in liver tumors displayed higher expression of exhausted genes including *Ctla4* and *Tnfrsf18* (Figure 3F). Furthermore, changes in CD8^+^ Tex proportions in SubQ‐only mice induced by PD‐1 blockade were absent in CSLT mice (Figure 3G). In summary, our data indicated that liver tumors could suppress the functional states and early Tex cells and might attenuate their responses to PD‐1 blockade, while the exact mechanisms still need further investigation.

### 2.5 Liver Tumors Impacted CD4^+^ T Cell Responses to Anti‐PD‐1 in mLNs

Since the T cell trafficking process from tumor‐draining LNs to the TME has been reported,^[^ ^26^ ^]^ we next examined the cellular relationship of tumor sites and mLNs, and whether liver tumors would impact immune cells in mLNs. While the proportions of CD8^+^ T cell subsets showed little changes among different conditions in mLNs, the fractions of CD4^+^ T cell subsets in mLNs were dramatically affected by liver tumors. PD‐1 blockade increased the fractions of CD4‐c02‐Itgb1 and CD4‐c03‐Il21 in SubQ‐only mice (Figure 3H), in line with the previous notion that tumor‐draining LN could supply T cell pools in tumors.^[^ ^26^, ^38^ ^]^ However, in mLNs of CSLT mice, anti‐PD‐1 barely changed the proportions of CD4‐c02‐Itgb1 and CD4‐c03‐Il21 (Figure 3H), suggesting that liver tumors might influence the responses of Tcm and Tfh to PD‐1 blockade in mLNs. To further evaluate the effects of implanted liver tumors and anti‐PD‐1 treatment for each immune population, we applied two‐way ANOVA tests on the mLN‐enriched clusters, and observed that these two factors together impacted Tcm (CD4‐c02‐Itgb1) and Tfh (CD4‐c03‐Il21) (Figure 5F).

Further, we traced the transitional relationship of Tex cells in the two tumor sites based on TCR information and STARTRAC, a tool of clonotype tracking to define clonal expansion, migration, and developmental transition of T cells.^[^ ^17^ ^]^ STARTRAC analyses revealed transitional processes of the mLN‐enriched cells (CD4‐c02‐Itgb1 and CD4‐c03‐Il21) with other clusters in tumors. Specifically, CD4‐c02‐Itgb1 cells were linked to tumor‐enriched Tcm (CD4‐c05‐S1pr1) cells and *Ifng* ^+^ Th1 (CD4‐c06‐Bhlhe40) cells based on STARTRAC‐tran indices (Figure 3I). CD4‐c03‐Il21 cells and *Ifng* ^+^ Th1 populations also showed high pairwise STARTRAC‐tran indices (Figure 3I), consistent with the notion that Th1‐like cells in tumors were developmentally linked to Tfh cells in LNs.^[^ ^39^ ^]^

### 2.6 Distinct Responses of Immunosuppressive Macrophage Subsets to Anti‐PD‐1 in Liver Tumors

**Figure 4:**
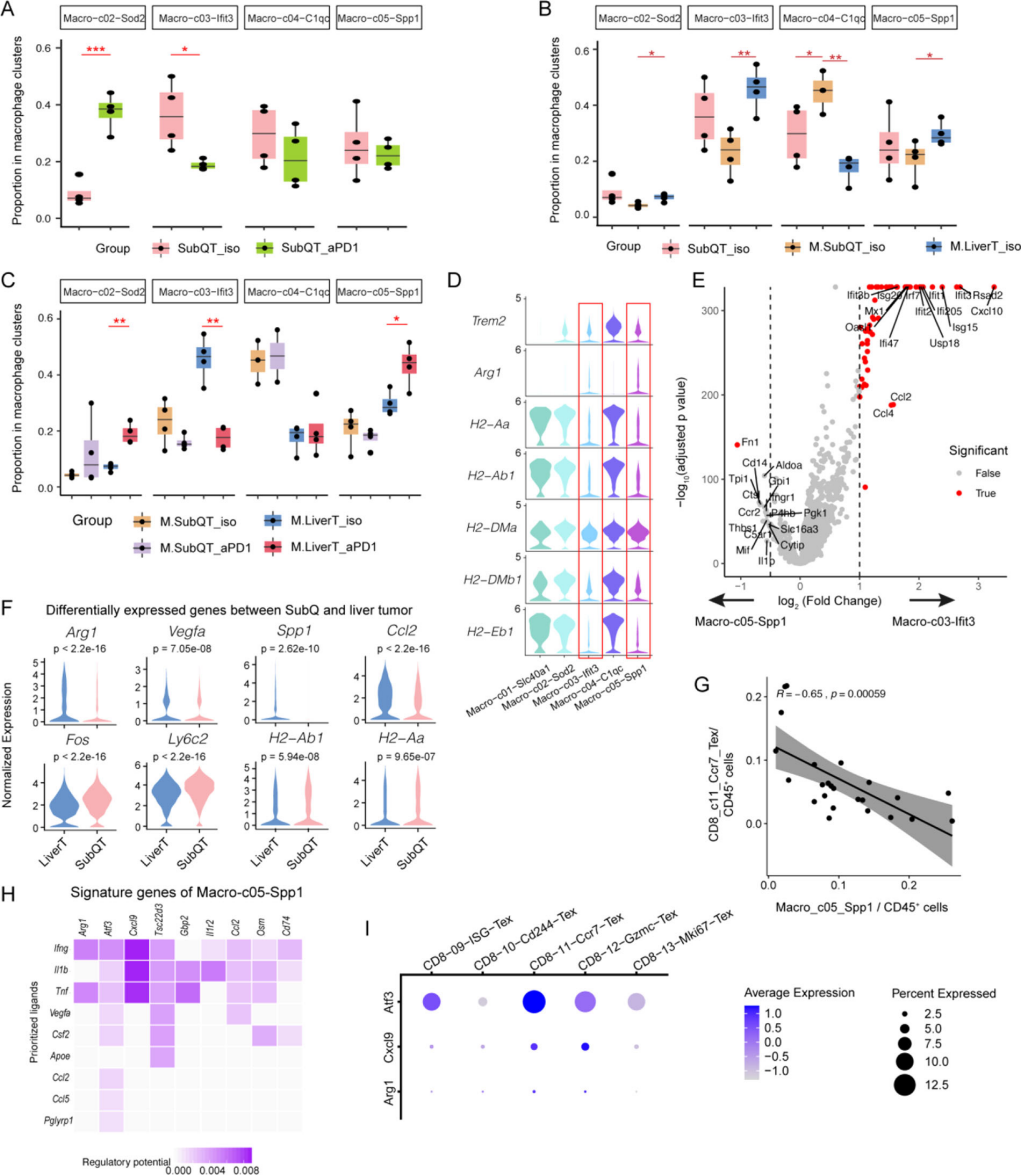
Alterations of macrophage subsets in different conditions including implanted liver tumors and PD‐1 blockade. A) Boxplots showing that PD‐1 blockade induced changes in the abundance of Macro‐02‐Sod2 and the reduction of Macro‐03‐Ifit3. *p < 0.05; **p < 0.01; ***p < 0.001. One‐sided *t*‐test. B) Boxplots showing the increased fractions of Macro‐03‐Ifit3 and Macro‐05‐Spp1 in liver tumors compared with the matched SubQ. *p < 0.05; **p < 0.01; ***, *p* < 0.001. One‐sided *t*‐test. C) Boxplots showing the changes of macrophage subsets induced by PD‐1 blockade in different tumor groups. *p < 0.05; **p < 0.01; ***p < 0.001. One‐sided *t*‐test. D) Violin plots showing similar expression features of Macro‐03‐Ifit3 and Macro‐05‐Spp1. E) Volcano plot showing the differentially expressed genes of Macro‐03‐Ifit3 and Macro‐05‐Spp1. F) Violin plots showing several genes differentially expressed on Macro‐05‐Spp1 in the two tumor sites. One‐sided *t*‐test. G) Scatter plots showing the frequency correlation of Macro‐05‐Spp1 with CD8_c11_Ccr7_Tex cells. H) Heatmap showing potential ligands driving the activation of Macro‐c05‐Spp1 predicted by NicheNet. I) Dot heatmap showing the expression of several receptors in Tex clusters. SubQT: subcutaneous tumors in SubQ‐only mice; M.SubQT: subcutaneous tumors in CSLT mice; M.LiverT: liver tumors in CSLT mice; iso: isotype; aPD1: anti‐PD‐1 treatment.

The transcriptomic heterogeneity and functional diversities of TAMs have recently been revealed in different cancer types of both humans^[^ ^18^, ^28^ ^]^ and mice.^[^ ^14^, ^25^, ^40^, ^41^ ^]^ We next sought to dissect how the implanted liver tumors would impact TAM populations in different sites and their responses to anti‐PD‐1 treatment. In SubQ‐only mice, anti‐PD‐1 increased the frequencies of Macro‐c02‐Sod2 but reduced the frequencies of Macro‐c03‐Ifit3 relative to those in treatment with a control antibody (**Figure** **4**A). Within CSLT mice, Macro‐c02‐Sod2, Macro‐c03‐Ifit3, and Macro‐c05‐Spp1 exhibited higher percentages of macrophages in liver tumors than in SubQ tumors, whereas the percentage of Macro‐c04‐C1qc was significantly reduced in liver tumors (Figure 4B). The elevation of Macro‐05‐Spp1 was consistent with the increased fraction of *SPP1* ^+^ macrophages in the liver metastasis of liver metastasized CRC patients.^[^ ^42^ ^]^ Although anti‐PD‐1 reduced total macrophage populations at both SubQ and liver tumors in CSLT mice, macrophage subsets displayed distinct responses to the treatment. While anti‐PD‐1 increased Macro‐c02‐Sod2 and decreased Macro‐c03‐Ifit3 fractions in liver tumors, similar to the observations in SubQ‐only mice, anti‐PD‐1 failed to modulate macrophage subsets frequencies in SubQ sites of CSLT mice (Figure 4C), which might hamper effective anti‐tumor response in their SubQ tumors. In particular, anti‐PD‐1 actually elevated Macro‐c05‐Spp1 frequencies in liver tumors (Figure 4C). By contrast, few alterations were observed in other myeloid populations including DC subsets in different conditions (Figure S5F, Supporting Information).

To understand how the aforementioned changes in TAM populations would impact the anti‐tumor immunity, we further investigated different expression signatures of these intra‐tumoral macrophage subsets. Macro‐c02‐Sod2 highly expressed T cell‐recruiting chemokines *Cxcl9* and *Cxcl10* (Figure S2E, Supporting Information), and was identical to a previously reported macrophage population in a CRC murine model that showed positive responses to ICB.^[^ ^43^ ^]^ In melanoma and lung cancer patients, high expression of *CXCL9* and *CXCL10* has also been reported to be associated with effective ICB.^[^ ^44^ ^]^ Macro‐c03‐Ifit3 and Macro‐c05‐Spp1 co‐expressed *Arg1* and *Trem2* (Figure 4D), markers of a recently reported suppressive myeloid population.^[^ ^41^ ^]^ Both of these two clusters also expressed low levels of *MHC* genes (Figure 4D), consistent with phenotypes of myeloid‐derived suppressor cell (MDSC) population.^[^ ^45^ ^]^ Macro‐c03‐Ifit3 exhibited high expression of interferon response genes like *Ifit2*, *Ifit3*, and *Isg15* (Figure 4E), indicating that MDSC‐like suppressive macrophages in tumors harbored further heterogeneity with distinct transcriptomic states. Furthermore, Macro‐c05‐Spp1 in liver tumors displayed more suppressive features than those in SubQ, with higher expression of immunosuppressive and angiogenic genes like *Cd274*, *Arg1*, *Spp1*, and *Vegfa*, and lower expression of inflammation and antigen‐presenting genes, including *Fos*, *Ly6c2*, *H2‐Eb1* and *H2‐Ab1* (Figure 4F and Table S3, Supporting Information).

We also noticed that the frequencies of Macro‐c05‐Spp1 were negatively correlated with several effector T cell subsets, including early pre‐exhausted Tex subset CD8‐c11‐Ccr7, *Ifng* ^+^ Th1 (CD4‐c06‐Bhlhe40), *Gzmk* ^+^ Tem (CD8‐c07‐Gzmk), and granzyme producing CD8‐c12‐Gzmc (Figure 4G and Figure S5H, Supporting Information). To further investigate the interaction relationship of Macro‐c05‐Spp1 and T cell subsets, we performed NicheNet and predicted a list of ligands that are functional in this macrophage population (Figure 4H). By examining the expression of corresponding receptors in Tex clusters, we found that the early exhausted populations showed the highest expression (Figure 4I), implicating the connection of these two populations where angiogenic macrophages might suppress the function of early exhausted T cells. In addition, applying the signatures of TAM subsets to TCGA CRC cohorts, we observed that the ratio of immunosuppressive macrophages (Macro‐c03‐Ifit3 and Macro‐c05‐Spp1) versus *Cxcl9^+^Cxcl10^+^* macrophages (Macro‐c02‐Cxcl9) was associated with poor prognosis (Figure S5I, Supporting Information). Altogether, our data suggested that tumors growing in the liver, which exhibited an immune tolerant environment, might increase fractions of pro‐tumor macrophage subsets including Macro‐c05‐Spp1 and Macro‐c03‐Ifit3 and impact their responses to PD‐1 blockade in multiple tumor sites of different organs.

### 2.7 Liver Tumors Modulated Responses of Interferon‐Stimulated Genes‐Expressing Populations to Anti‐PD1

**Figure 5:**
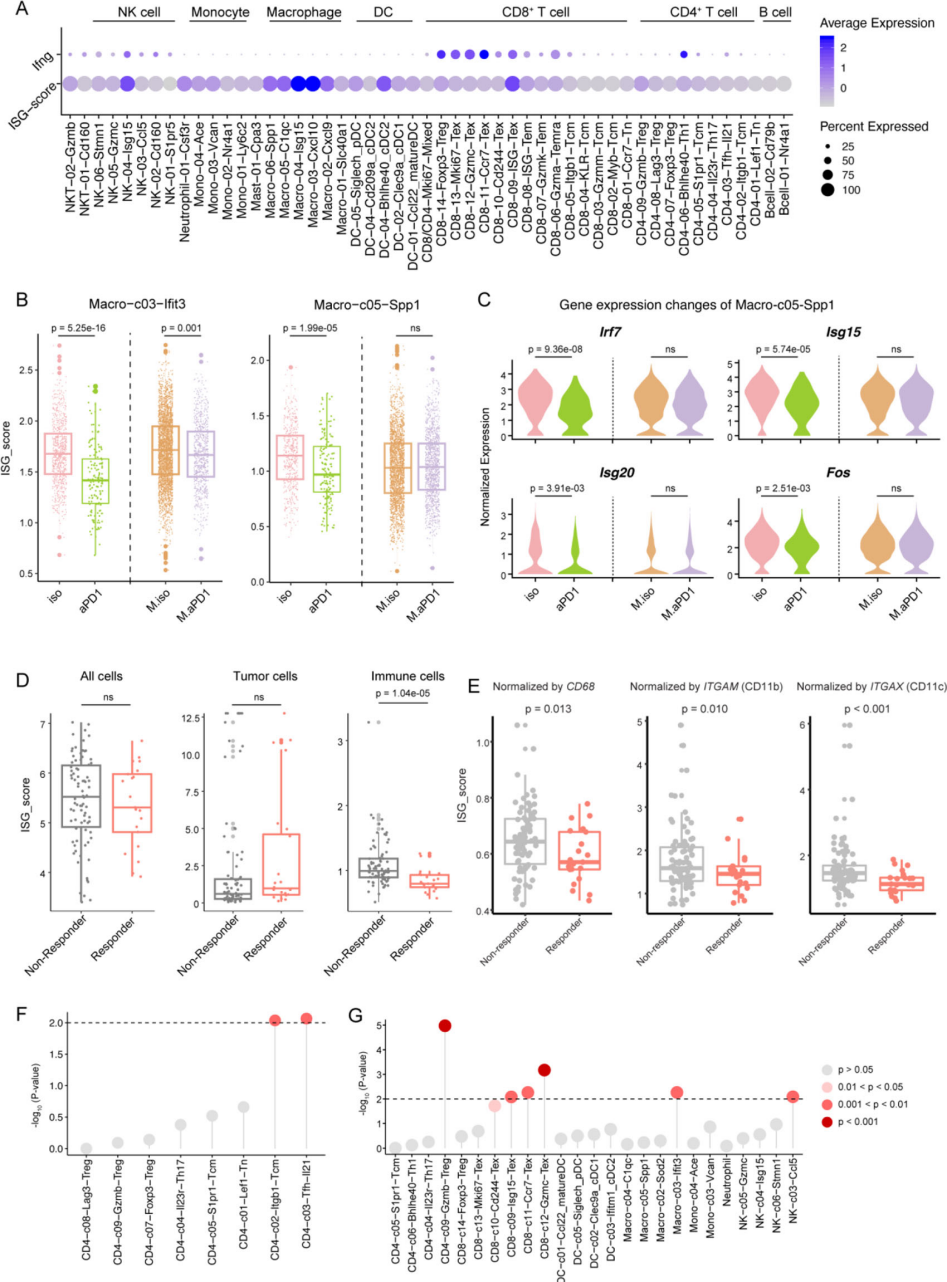
Interferon signaling‐related alterations in different treatment groups impacted by liver tumors. A) Dot heatmap showing the expression of *Ifng* and the ISG score in all the immune cell clusters. B) Boxplots showing the ISG score changes of Macro‐03‐Ifit3 and Macro‐05‐Spp1 in different treatment groups. One‐sided *t*‐test. C) Violin plots showing the molecular changes of Macro‐05‐Spp1 in different treatment groups. One‐sided *t*‐test. D,E) Boxplots showing the ISG scores of different cell populations in responders and non‐responders of melanoma patients treated with anti‐PD‐1.^[^ ^48^ ^]^ D) The cell populations include the whole populations, tumor cells (normalized by *EPCAM*), immune cells (normalized by *PTPRC*), and E) different myeloid populations (normalized by *CD68*, *ITGAM*, and *ITGAX*). Dot plot showing the interaction *p*‐value of implanted liver tumors and anti‐PD‐1 treatment for F) mLN‐ and G) tumor‐enriched clusters by ANOVA analyses. Each dot represents a cell cluster. Dash line represents *p* = 0.01. iso: isotype in SubQ‐only mice; aPD1: anti‐PD‐1 treatment in SubQ‐only mice; M.iso: isotype in CSLT mice; M.aPD1: anti‐PD‐1 treatment in CSLT mice.

Having characterized variations of different immune populations, we next sought to interrogate their common expression features. Three clusters—CD8‐c09‐Isg15, NK‐c04‐Isg15, and Macro‐c03‐Ifit3 were characterized by high expression levels of interferon‐stimulated genes (ISGs) such as *Ifit2*, *Ifit3*, and *Isg15*. Since *Ifng* instead of type I interferon genes were detected in our data (**Figure** **5**A), we reasoned that the ISGs in TME were mainly induced by type II interferon signaling. Specifically, the expression of *Ifng* was detected in Th1‐like CD4^+^, CD8^+^ Tex, and NK cell subsets. Comparing the ISG scores based on the gene set generated by Benci et al.^[^ ^46^ ^]^ among different clusters, we observed that Macro‐c03‐Ifit3, CD8‐c09‐Isg15, and NK‐c04‐Isg15 showed the top 3 highest ISG scores(Figure 5A). In addition, TAM and cDC2 clusters exhibited higher ISG scores than lymphocytes, suggesting that myeloid cells might be more sensitive to interferon signaling. Applying NicheNet analysis on the clusters, we indeed observed *Ifng* as top ligand activating different ISG‐expressing populations including Macro‐c03‐Ifit3, Macro‐c05‐Spp1, and CD8‐c09‐Isg15 (Figure S8G,H, Supporting Information).

It has been reported that prolonged ISG expression in cancer cells increased ICB resistance in mice,^[^ ^47^ ^]^ while blocking tumor interferon signaling improved tumor‐killing processes of lymphocytes (T, NK cells, and ILCs).^[^ ^46^ ^]^ However, the relationships of ISG‐expressing immune populations and their responses to ICB have not been fully understood. Of note, all three ISG^hi^ populations (CD8‐c09‐Isg15, NK‐c04‐Isg15, and Macro‐c03‐Ifit3) showed frequency reduction following anti‐PD‐1 treatments in SubQ‐only mice, whereas such tendencies were not observed in SubQ tumors of CSLT mice, indicating that liver tumors might impact the responses of ISG^hi^ immune populations to PD‐1 blockade. In SubQ‐only mice, we observed significant reductions of ISG scores in Macro‐c03‐Ifit3 and Macro‐c05‐Spp1 induced by anti‐PD‐1, while in CSLT mice, such tendencies were eliminated (Figure 5B). ISG genes individually also exhibited similar tendencies in Macro‐c05‐Spp1 (Figure 5C). By comparison, CD8‐c09‐Isg15 and NK‐c04‐Isg15 did not show significant changes in either SubQ‐only or CSLT mice (Figure S8E, Supporting Information). Such observations suggested that in addition to tumor cells that have already been reported,^[^ ^45^ ^]^ these immunosuppressive TAMs might also correlate with ICB resistance.

To extrapolate these observations and their potential clinical investigation to patients, we next examined ISG scores in a melanoma cohort following PD‐1 treatment.^[^ ^48^ ^]^ Although global ISGs expression and ISG scores of cancer cells (normalized by *EPCAM*) did not show differences between responders and non‐responders, ISG scores of immune cell populations (normalized by *PTPRC*) in responders were much lower than those in non‐responders (Figure 5D), confirming that prolonged ISGs in immune cell populations, especially in myeloid cells (Figure 5E), might be associated with ICB resistance. In addition, when normalized by features of Macro‐c03‐Ifit3 or Macro‐c05‐Spp1, responders also showed lower ISG scores than non‐responders (Figure S8F, Supporting Information). Furthermore, ANOVA tests on the tumor‐enriched clusters revealed that CD8‐c09‐Isg15, CD8‐c10‐Cd244, CD8‐c11‐Ccr7, CD8‐c12‐Gzmc, CD4‐c09‐Gzmb, Macro‐c03‐Ifit3, and NK‐c03‐Ccl5 were impacted by combined effects of liver tumors and anti‐PD‐1 treatment (Figure 5G). Taken together, the alterations of these populations in mice might partially explain the immune resistance in liver metastasized CRC patients, while further investigations are still needed.

## 3 Conclusion

Based on the murine models, we systematically studied changes in different tumor sites and different treatment groups to unveil the effects of liver metastasis on immunotherapy response. Our data revealed that liver tumors were associated with multiple immunosuppressive alterations including frequencies and molecular changes of Tex and TAM subsets. The diminished responses to anti‐PD‐1 treatment in CSLT mice demonstrated that liver metastasis could trigger systematic effects on responses to ICB. Notably, while anti‐PD‐1 did not cause frequency alterations of angiogenic TAMs in SubQ tumors in either SubQ‐only or CSLT mice, the treatment dramatically elevated angiogenic TAMs frequencies in liver tumors. In addition, alterations of CD8^+^ Tex proportions in SubQ‐only mice induced by PD‐1 blockade were absent in CSLT mice, suggesting that in metastatic tumors, the anti‐PD‐1 treatment might actually entice the tumor‐promoting effects of TME. With the systematic comparison of human and mouse datasets, we observed that the immune cell populations in the two species were largely conserved, in line with previous studies.^[^ ^25^ ^]^ Thus, our observations in mice may have clinical application potential. The reduction of ISG expression in myeloid cells might be associated with effective responses to anti‐PD‐1 treatment, while liver tumors hampered the alterations of ISG expression. Since the expression of ISGs on immune cells in responders was higher than those in non‐responders, these genes may serve as potential biomarkers for patient stratification. However, our study only characterized the ISGs expression at the endpoint and it is worthwhile to trace the ISG alterations at different time points following metastasis or treatments. In addition, the exact mechanism of the relationship of liver metastasis and immunotherapy efficacy warrants further investigation.

Currently, although our CSLT model is not the ideal liver metastasis model as metastatic tumor migration processes to the liver are not spontaneous, it could still somehow reflect liver metastasis mechanisms in CRC liver metastasis patients.^[^ ^41^ ^]^ For instance, we observed that in both mouse and patient samples, the frequencies of myeloid cells including monocyte, DCs, and macrophages showed increases in metastatic tumors compared with primary tumors. Regardless, our CSLT model could only study the impact of the cooccurrence of liver tumor on the efficacy of PD‐1 blockade tumors in other organs but not *de novo* liver metastasis, and other optimal models recapitulating the complete tumor migration processes from the primary tumors still need further exploration. In summary, our study characterized detailed immune‐alterations associated with liver metastases and PD‐1 blockade at the transcription level. Our findings complement the growing clinical studies that have demonstrated a reduced response to ICBs in cancer patients with liver metastases, and implicate additional regulatory factors including TAMs and Tex populations that are associated with liver tumors. Although the models may not fully represent human pathophysiology, our work provides insights into important determinants of immunotherapy efficacy, which can echo back to clinical observations. Further treatment strategies need to be developed to complement the current PD‐1 blockade paradigm, in order to overcome the complex immune‐suppressive patterns associated with liver metastases.

## 4 Experimental Section

### Hemi‐Spleen Removal Surgery of Murine Models

After anesthesia administration, a small incision (≈1 cm) was made in the skin of the abdominal wall on the left flank then the spleen was carefully exposed. After exposing the spleen, the splenic blood vessels were located at the inferior end of the spleen. Then two medium size ligating clips were placed in the center of the spleen to divide the spleen, being careful to avoid any trauma of the splenic vessels at the end of the splenic poles. After the spleen was divided the upper pole of the spleen was placed back into the abdominal cavity. 50 µL of cell suspension was injected slowly into the exposed hemi‐spleen using a 30 G needle. 3 to 5 min after the injection was completed a medium size ligating clip was placed under the spleen to the most distal aspect of the splenic vessels. Then a ligature using absorbable suture (4‐0) was placed around the splenic blood vessels. Following this step, the hemi‐spleen was removed. The procedure was according to Soares et al.^[^ ^29^ ^]^ Then the abdominal incision was closed with absorbable suture (4‐0), and the borders of the skin incision were brought together and closed with wound clips. The wound clips were removed within 10 to 14 days after placement. The first dose of Buprenorphine was given after the animal was induced with anesthesia and before the first surgical incision was made. One dose of Buprenorphine was given right after the surgical procedure was performed. Post‐surgery analgesia was administered as needed if the animal exhibits signs of pain (2×/daily (Q 10–12 h.) for up to 3 days). Mice will be monitored twice daily for 5 days followed by once daily for an additional 2–4 days.

### Intra‐Splenic Injection with Subcutaneous Tumor Model

The luciferase labeled murine colorectal line MC38‐Luc was injected intra‐splenic (5 × 10^5^/mouse), 48 h post‐operative period 3 × 10^5^ MC38 cells were injected subcutaneously (s.c.) into the lower right flank of 6–8‐week‐old female C57BL/6 mice (Charles River Lab). 12 days after the s.c. injection, in vivo bioluminescence (BLI) was measured at region of liver area using IVIS Spectrum (Xenogen Corp) to determine establishment of liver metastasis. Prior to imaging, mice were injected with 150 mg kg^–1^ luciferin, IP. Animals were randomized into groups (*N* = 10 per group) such that the average tumor volume and BLI signal at the beginning of treatment administration were uniform across treatment groups. Animals were then IP administrated murine IgG1 antibody control or murine PD‐1 antibody every 3 days for three doses total. Clinical signs, body weight changes, and tumor growth rate were measured twice per week until study termination. To generate survival graph, animals were taken down once their tumor burden reached 800 mm^3^. For single cell RNA‐seq study, all animals were euthanized on day 22 post hemi‐spleen removal surgery. All animal experiments were conducted with approval by the Amgen Institutional Animal Care and Use Committee.

### Tissue Dissociation

Subcutaneous tumors and liver metastatic tumors were dissociated into single cell suspensions as previously described.^[^ ^36^ ^]^ Briefly, tumors were minced and resuspended in DMEM/F12 supplemented with Liberase TL (Roche) and DNase I followed by mechanical dissociation and straining through 70 µm filters. Cells were counted on the ViCell XR (Beckman Coulter) prior to enrichment for total CD45+ cells using the Invitrogen Mouse CD45 positive selection kit (Invitrogen 8802‐6865‐74).

Normal liver samples from non‐metastatic mice were first perfused with PBS prior to mincing and treatment with DNAse/Liberase under same conditions as the tumors. Spleen and subcutaneous draining lymph nodes were mechanically homogenized and strained through 70 µm filter.

### Single Cell Sorting, Library Preparation, and Sequencing

Single cell suspensions for all samples were stained for bulk sorting prior to single cell processing. Liver metastatic tumors, subcutaneous tumor, and normal liver samples were stained with CD45‐BV510 (BD) and viability dye (Sytox red Fisher S34859) and sorted for live total CD45+ cells. mLN samples were stained with a‐TCRb PE (BD CD44 BV421 and viability dye (Sytox Red) followed by sorting for viable TCR^+^CD44^+^ cells. Two populations were sorted from spleen samples: CD44+ T cells as in mLN samples, and a second sort for CD45+CD19‐TCR‐ splenic myeloid cells.

Sorted cells were centrifuged at 4 degrees and resuspended in a volume of 200 µL PBS without BSA. Individual samples were then labeled with lipid‐modified oligos (LMO) as described in McGinnis et al. Nat. Methods 2019 with minor modifications. Biological replicates were incubated with a unique LMO followed by addition of lipid co‐anchor. Cells were washed twice with ice‐cold PBS + 1% BSA twice prior to pooling of samples. Pooled samples were then encapsulated with the 10× Genomics 5′ VDJ kit according to manufacturer specifications.

Single cell gene expression and TCR libraries were generated according to manufacturer protocol with the modification of supplementing the cDNA amplification PCR step with 1 µL of 2.5 uM LMO primer (CTTGGCACCCGAGAATTCC). LMO hashing libraries were made as described in McGinnis et al. Nat. Methods 2019. Libraries were sequenced on NovaSeq S4.

Single‐cell RNA‐seq data processing: For scRNA‐seq data, the Cell Ranger toolkit (version 3.0.0) provided by 10× Genomics was applied to generate the gene‐cell unique molecular identifier (UMI) matrix, with GRCm38 as reference genome. Genes of the expression matrix were filtered out if they were expressed in less than 10 cells. The quality control criteria for cells were that the detected gene number for each cell was ≈600–6000, and the percentage of mitochondrial gene expression was less than 0.125. Such a high threshold was used to ensure that the most of barcodes associated with empty partitions or doublet cells were filtered out. After the quality control process, 91 902 cells with 16 029 detected genes were obtained for downstream analysis.

To demultiplex cells to their original sample‐of‐origin, cell hashing was performed in Seurat as previously reported. In the 91 902 cells, 38 222 singlets (cells that were positive for only one LMO) were detected with one sample origin, while others were detected with none or more than one LMO.

### Unsupervised Clustering Analysis and Dimension Reduction

Unsupervised clustering analyses were performed by the Louvain algorithm in Scanpy. Specifically, the highly variable genes were generated with appropriate thresholds of the mean expression and dispersion (variance/mean). Principal component analysis (PCA) was performed on ≈1000–2000 variable genes. Louvain analysis was performed on 40 PCs with resolution 1.5 to perform the first‐round cluster and annotated each cluster by known markers. Ten major cell types, including 5 lymphoid cell clusters (CD4^+^ T, CD8^+^ T, NKT, NK, and B cells) and 5 myeloid cell clusters (monocytes, macrophages, DCs, mast cells, and neutrophils), were identified after the first‐round clustering. The second‐round clustering was performed according to the same range of parameters to identify clusters within the major cell types (T cells, myeloid cells, NK cells, and B cells) aforementioned. For visualization, the dimensionality of the dataset was reduced by UMAP. To identify the differentially expressed genes for each cluster, the dataset was transformed to Seurat object, and *FindAllMarker* function was used to perform the calculation. The Wilcoxon test was used for each cluster against all the other cells.

### Tissue Preference Based on Ro/e

To quantify the preference of each cluster across tissues, the observed and expected cell numbers in each cluster were compared. The expected cell numbers for each combination of cell clusters and tissues were obtained from the chi‐squared test. One cluster was identified as being enriched in a specific tissue if Ro/e > 1. The cluster preference in a specific tissue was defined based on Ro/e. +++, Ro/e > 3; ++, 1 <Ro/e ≤ 3; +, 0.2 ≤ Ro/e ≤ 1; +/−, 0 < Ro/e < 0.2; −, Ro/e = 0.

### TCR Analysis

The TCR sequences for each single cell were processed using Cell Ranger (version 2.1.0) against the manufacturer‐supplied mouse vdj reference genome. In all TCR contigs assembled, the low‐confidence, non‐productive, or those UMIs < 2 were first discarded. For cells with two or more α or β chains assembled, the α–β pair showing the highest expression level (UMI) was defined as the dominant α–β pair in the corresponding cell. Each unique dominant alpha–beta pair was defined as a clonotype. If one clonotype was present in at least two cells, this clonotype would be considered clonal, and the number of cells with such a dominant alpha–beta pair indicated the degree of clonality of the clonotype. The TCR alpha–beta pairs for 13 141 CD4^+^ T cells and 16 338 CD8^+^ T cells were identified, of which 4626 and 6523 CD4^+^ and CD8^+^ T cells could be traced to sample origins.

### Similarity Analysis of Clusters from Human and Mouse

To systematically link cell clusters from human and mouse, the SciBet method was used to examine the similarity of cells across species. After selecting marker genes with E‐test (*k* = 50), the dataset was set as the reference set, and data from Zhang et al.^[^ ^4^ ^]^ were set as the query set. Confusion heat‐maps generated by SciBet suggested cell clusters in humans and mice were largely conserved.

### Developmental Trajectory Inference

To infer the developmental relationship of exhausted CD8^+^ T cell clusters, monocle3 was performed on the dataset. Five Tex clusters were selected, and the parameters were set as defaulted.

### Survival Analysis

The TCGA COAD (Colon adenocarcinoma) data were used to test the correlation of selected genes and patient survival. The gene expression data and the clinical data were downloaded from UCSC Xena (http://xena.ucsc.edu/). The feature genes used for cell clusters during analyses were based on differentially expressed genes (FDR < 0.01, log_2_(Fold Change) > 2) of the cluster versus other subsets in the same major cell type. The statistical analysis was performed by GEPIA2.

## Conflict of Interest

S.L., D.B., J.E., B.B., and W.O. are employees of Amgen Inc. Other authors declare no conflict of interest.

## Supporting information

Supporting Information

SupplementaryTable 1

SupplementaryTable 2

SupplementaryTable 3

SupplementaryTable 4

SupplementaryTable 5

SupplementaryTable 6

## Data Availability

The processed gene expression data of this study can be obtained from Gene Expression Omnibus (GEO) with an accession number of GSE164522. The raw FASTQ files in this study will be provided for scientific research upon request.
