# Supplementary material for: Liver Metastasis Modulate Responses of Suppressive Macrophages and Exhausted T Cells to Immunotherapy Revealed by Single Cell Sequencing
Source: Adv Genet (Hoboken). 2022 Oct 11;3(4):2200002. doi: 10.1002/ggn2.202200002 (PMC9993474; doi:10.1002/ggn2.202200002)
Supplement: Supplementary file 1 — Supporting Information [file GGN2-3-2200002-s005.pdf]

ADVANCED GENETICS
Open Access

Supporting Information

for *Advanced Genetics*, DOI 10.1002/ggn2.202200002

Liver Metastasis Modulate Responses of Suppressive Macrophages and Exhausted T Cells to Immunotherapy Revealed by Single Cell Sequencing

Qiming Zhang, Siyuan Liu, Yedan Liu, Dev Bhatt, Juan Estrada, Brian Belmontes, Xianwen Ren, Jude Canon* and Wenjun Ouyang**

Supporting Information

Liver metastasis modulate responses of suppressive macrophages and exhausted T cells to immunotherapy revealed by single cell sequencing

Qiming Zhang, Siyuan Liu, Yedan Liu, Dev Bhatt, Juan Estrada, Brian Belmontes, Xianwen Ren, Zemin Zhang, and Jude Canon, Wenjun Ouyang**

Supplemental methods

Hemi-spleen removal surgery of murine models

After anesthesia administration, a small incision (~1cm) is made in the skin of the abdominal wall on the left flank then the spleen is carefully exposed. After exposing the spleen, the splenic blood vessels are located at the inferior end of the spleen. Then two medium size ligating clips are placed in the center of the spleen to divide the spleen, being careful to avoid any trauma of the splenic vessels at the end of the splenic poles. After the spleen is divided the upper pole of the spleen is placed back into the abdominal cavity. 50 μL of cell suspension is injected slowly into the exposed hemi-spleen using a 30 G needle. 3 to 5 minutes after the injection is completed a medium size ligating clip is placed under the spleen to the most distal aspect of the splenic vessels. Then a ligature using absorbable suture (4-0) is placed around the splenic blood vessels. Following this step, the hemi-spleen is removed. The procedure is according to Soares et al. Then the abdominal incision is closed with absorbable suture (4-0), and the borders of the skin incision are brought together and closed with wound clips. The wound clips are removed within 10 to 14 days after placement. The first dose of Buprenorphine is given after the animal is induced with anesthesia and before the first surgical incision is made. One dose of Buprenorphine is given right after the surgical procedure is performed. Post-surgery analgesia is administered as needed if the animal exhibits signs of pain (2x/daily (Q 10-12 hrs.) for up to 3 days). Mice will be monitored twice daily for 5 days followed by once daily for an additional 2-4 days.

Intra-splenic injection with subcutaneous tumor model

The luciferase labeled murine colorectal line MC38-Luc was injected intra-splenic (5 x 10^5/mouse), 48 hrs post-operative period 3 x 10^5 MC38 cells were injected subcutaneously (s.c.) into the lower right flank of 6-8-week-old female C57BL/6 mice (Charles River Lab). 12 days after the s.c. injection, in vivo bioluminescence (BLI) was measured at region of liver area using IVIS Spectrum (Xenogen Corp) to determine establishment of liver metastasis. Prior to imaging, mice were injected with 150 mg kg^{-1} luciferin, IP. Animals were randomized into groups (N = 10 per group) such that the average tumor volume and BLI signal at the beginning of treatment administration was uniform across treatment groups.

Animals were then IP administrated murine IgG1 antibody control or murine PD-1 antibody every three days for three doses total. Clinical signs, body weight changes, and tumor growth rate were measured twice per week until study termination. To generate survival graph, animals were taken down once their tumor burden reached 800mm. For single cell RNA-seq study, all animals were euthanized on day 22 post hemi-spleen removal surgery. All animal experiments were conducted with approval by the Amgen Institutional Animal Care and Use Committee.

Tissue dissociation process

Subcutaneous tumors and liver metastatic tumors were minced into 1 mm pieces and resuspended in 10 mL of DMEM/F12 supplemented with 200 μg/mL Liberase TL (Roche) and DNase I 5 μg/mL (Sigma) followed by mechanical dissociation using Miltenyi C-tubes and the gentleMACS dissociater with the manufacturer program hu_tumor_01. Suspensions were incubated at 37°C with shaking at 180 rpm for 20 minutes, followed by enzyme quenching by addition of 1 mL FBS and additional two rounds of gentleMACS dissociation using program hu_tumor_02. Tumor suspensions were strained through 70 μm filters. The filter was rinsed with 10 mL of MACS buffer (2% FBS + 2 mM EDTA). Cells were then pelleted at 1500 rpm for 5 minutes at 4°C and resuspend in 5 mL of MACS buffer. Cells were counted on the ViCell XR (Beckman Coulter) prior to enrichment for total $CD45^+$ cells using the Invitrogen Mouse CD45 positive selection kit (Invitrogen 8802-6865-74).

Normal liver samples from non-metastatic mice were first perfused with PBS prior to mincing and treatment with DNAse/Liberase under same conditions as the tumors. Spleen and subcutaneous draining lymph nodes were mechanically homogenized and strained through 70 μM filter.

Single cell sorting, library preparation and sequencing

Single cell suspensions for all samples were stained for bulk sorting prior to single cell processing. Liver metastatic tumors, subcutaneous tumor, and normal liver samples were stained with CD45-BV510 (BD) and viability dye (Sytox red Fisher S34859) and sorted for

live total CD45$^+$ cells. mLN samples were stained with a-TCRb PE (BD CD44 BV421 and viability dye (Sytox Red) followed by sorting for viable TCR$^+$CD44$^+$ cells. Two populations were sorted from spleen samples: CD44$^+$ T cells as in mLN samples, and a second sort for CD45$^+$CD19$^-$TCR$^-$ splenic myeloid cells.

Sorted cells were centrifuged at 4 degrees and resuspended in a volume of 200 µL PBS without BSA. Individual samples were then labelled with lipid-modified oligos (LMO) as described in McGinnis et. al. Nat. Methods 2019 with minor modifications. Briefly, LMOs were made into a 2uM equimolar mix with individual oligo barcodes of the sequence format CCTTGGCACCCGAGAATTCCA-xxxxxxxxxxxxxxx-CCCATATAAGAAA with the x's representing unique hashing barcodes. 20 µL of the LMO:barcode mix was incubated with sorted cells for 5 minutes on ice, followed by addition of 20 µL of a 2 µM co-anchor lipid for another 5 minute incubation. Cells were washed twice with ice-cold PBS + 1% BSA twice prior to pooling of samples. Pooled samples were then centrifuged and resuspended to a target concentration of 5 x 10^5 - 1 x 10^6 cells/ml prior to encapsulation with the 10x Genomics 5' VDJ kit.

Single cell gene expression and TCR libraries were generated according to manufacturer protocol with the modification of supplementing the cDNA amplification PCR step with 1 µL of 2.5uM LMO primer (CTTGGCACCCGAGAATTCC) and retaining the supernatant of the cDNA amplification cleanup step for generation of LMO hashing libraries as described in McGinnis et. al. Nat. Methods 2019. Libraries were quantified on the Agilent Tapestation and pooled equimolar at a ration of 10:1:1 of gene expression:TCR:LMO libraries. Samples were sequenced on NovaSeq S4 lanes targeting a read depth of 50000 reads/cell according to manufacturer recommended specifications.

Single-cell RNA-seq data processing

For scRNA-seq data, the *Cell Ranger* toolkit (version 3.0.0) provided by 10x Genomics was applied to generate the gene-cell unique molecular identifier (UMI) matrix, with GRCm38 as reference genome. Genes of the expression matrix were filtered out if they were expressed in less than 10 cells. The quality control criteria for cells were that the detected gene number for each cell was in 600~6000, and the percentage of mitochondrial gene expression was less than

0.125. We used such a high threshold to ensure that we have filtered out the most of barcodes associated with empty partitions or doublet cells. After the quality control process, we obtained 91,902 cells with 16,029 detected genes for downstream analysis.

To demultiplex cells to their original sample-of-origin, we performed Cell Hashing in Seurat as previously reported.[1] In the 91,902 cells, 38,222 singlets (cells that were positive for only one LMO) were detected with one sample origin, while others were detected none or more than one LMO.

Unsupervised clustering analysis and dimension reduction

We performed unsupervised clustering analyses by the louvain algorithm[2] in scanpy.[3] Specifically, the highly variable genes were generated with appropriate thresholds of the mean expression and dispersion (variance/mean). Principal component analysis (PCA) was performed on 1000~2000 variable genes. We performed louvain analysis on 40 PCs with resolution 1.5 to perform the first-round cluster and annotated each cluster by known markers. Ten major cell types, including 5 lymphoid cell clusters ($CD4^+$ T, $CD8^+$ T, NKT, NK and B cells) and 5 myeloid cell clusters (monocytes, macrophages, DCs, mast cells and neutrophils), were identified after the first-round clustering. The second-round clustering was performed according to the same range of parameters to identify clusters within the major cell types (T cells, myeloid cells, NK cells and B cells) aforementioned.

For visualization, the dimensionality of our dataset was reduced by UMAP.

To identify the differentially expressed genes for each cluster, we transformed our dataset to seurat object, and used *FindAllMarker* function to perform the calculation. The wilcoxon test was used for each cluster against all the other cells.

Tissue preference based on Ro/e

To quantify the preference of each cluster across tissues, we compared the observed and expected cell numbers in each cluster as we described previously.[4] The expected cell numbers for each combination of cell clusters and tissues were obtained from the chi-squared

test. One cluster was identified as being enriched in a specific tissue if $Ro/e > 1$. We defined the cluster preference in a specific tissue based on Ro/e. +++, $Ro/e > 3$; ++, $1 < Ro/e \leq 3$; +, $0.2 \leq Ro/e \leq 1$; +/−, $0 < Ro/e < 0.2$; −, $Ro/e = 0$.

TCR analysis

The TCR sequences for each single cell were processed using Cell Ranger (version 2.1.0) against the manufacturer-supplied mouse vdj reference genome. In all TCR contigs assembled, we first discarded the low-confidence, non-productive or those UMIs < 2. For cells with two or more α or β chains assembled, the α-β pair showing the highest expression level (UMI) was defined as the dominant α-β pair in the corresponding cell. Each unique dominant alpha-beta pair was defined as a clonotype. If one clonotype was present in at least two cells, this clonotype would be considered clonal, and the number of cells with such a dominant alpha-beta pair indicated the degree of clonality of the clonotype. We identified the TCR alpha-beta pairs for 13,141 CD4$^+$ T cells and 16,338 CD8$^+$ T cells, of which 4,626 and 6,523 CD4$^+$ and CD8$^+$ T cells could be traced to sample origins.

Similarity analysis of clusters from human and mouse

To systematically link cell clusters from human and mouse, we used the SciBet method[5] to examine the similarity of cells across species. After selecting marker genes with E-test ($k = 50$), we set our dataset as the reference set and data from Zhang et al.[4] as the query set. Confusion heat-maps generated by SciBet suggested cell clusters in humans and mice were largely conserved.

Developmental trajectory inference

To infer the developmental relationship of exhausted CD8$^+$ T cell clusters, we performed monocle3[6] on our dataset. Five Tex clusters were selected, and the parameters were set as defaulted.

Survival analysis

The TCGA COAD (Colon adenocarcinoma) data were used to test the correlation of selected genes and patient survival. The gene expression data and the clinical data were downloaded from UCSC Xena (http://xena.ucsc.edu/). The feature genes used for cell clusters during analyses were based on differentially expressed genes (FDR < 0.01, $\log_2$(Fold Change) > 2) of the cluster versus other subsets in the same major cell type. The statistical analysis was performed by GEPIA2.[7]

References

[1] M. Stoeckius, S. Zheng, B. Houck-Loomis, S. Hao, B. Z. Yeung, W. M. Mauck 3rd, P. Smibert, R. Satija, *Genome Biol.* **2018**, *19*, 224.

[2] V. D. Blondel, J.-L. Guillaume, R. Lambiotte, E. Lefebvre, *Journal of Statistical Mechanics: Theory and Experiment* **2008**, *2008*, P10008.

[3] P. Savas, B. Virassamy, C. Ye, A. Salim, C. P. Mintoff, F. Caramia, R. Salgado, D. J. Byrne, Z. L. Teo, S. Dushyanthen, A. Byrne, L. Wein, S. J. Luen, C. Poliness, S. S. Nightingale, A. S. Skandarajah, D. E. Gyorki, C. M. Thornton, P. A. Beavis, S. B. Fox, Kathleen Cuningham Foundation Consortium for Research into Familial Breast Cancer (kConFab), P. K. Darcy, T. P. Speed, L. K. Mackay, P. J. Neeson, S. Loi, *Nat. Med.* **2018**, *24*, 986.

[4] L. Zhang, X. Yu, L. Zheng, Y. Zhang, Y. Li, Q. Fang, R. Gao, B. Kang, Q. Zhang, J. Y. Huang, H. Konno, X. Guo, Y. Ye, S. Gao, S. Wang, X. Hu, X. Ren, Z. Shen, W. Ouyang, Z. Zhang, *Nature* **2018**, *564*, 268.

[5] C. Li, B. Liu, B. Kang, Z. Liu, Y. Liu, C. Chen, X. Ren, Z. Zhang, *Nat. Commun.* **2020**, *11*, 1818.

[6] J. Cao, M. Spielmann, X. Qiu, X. Huang, D. M. Ibrahim, A. J. Hill, F. Zhang, S. Mundlos, L. Christiansen, F. J. Steemers, C. Trapnell, J. Shendure, *Nature* **2019**, *566*, 496.

[7] Z. Tang, B. Kang, C. Li, T. Chen, Z. Zhang, *Nucleic Acids Res.* **2019**, *47*, W556.

Supplemental methods

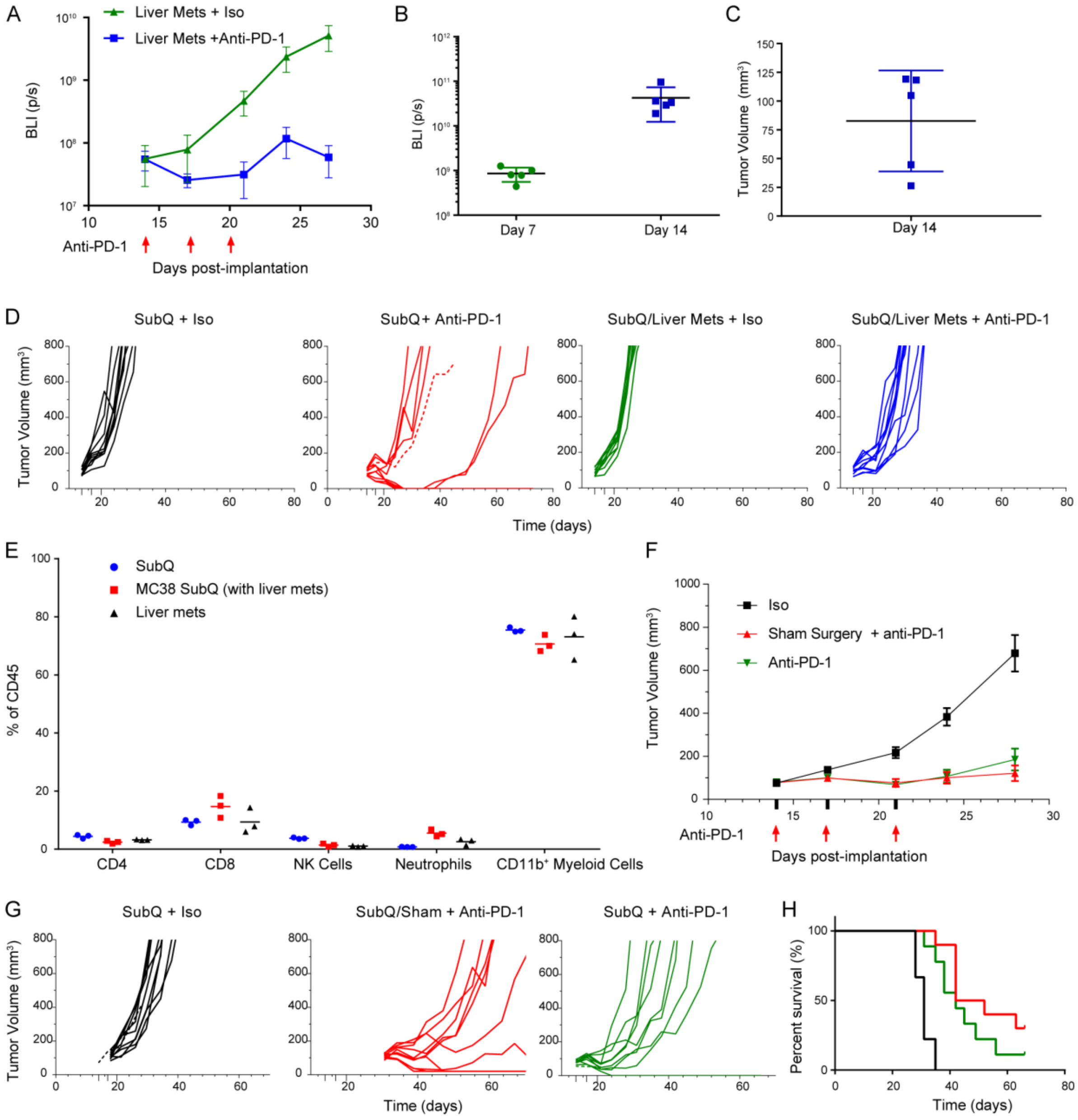

Figure S1. Reduced responses to anti-PD-1 in the concurrently subcutaneous and liver tumor (CSLT) murine models.

(A) Growth curves showing the responses of liver tumors to anti-PD-1 treatment.

(B) Dot plot showing tumor growth at day 7 and 14.

(C) Dot plot showing the tumor volume at day 14.

(D) SubQ tumor growth curves of different experimental groups including implanted liver tumors and anti-PD-1 treatment, shown in individual.

(E) Dot plot showing the proportions of major immune cell populations in different groups based on FACS.

(F) SubQ tumor growth curves showing that surgery did not influence the response to PD-1 blockade.

(G) SubQ tumor growth curves showing that surgery did not influence the response to PD-1 blockade, shown in individual.

(H) Survival curves showing that surgery did not influence the response to PD-1 blockade.

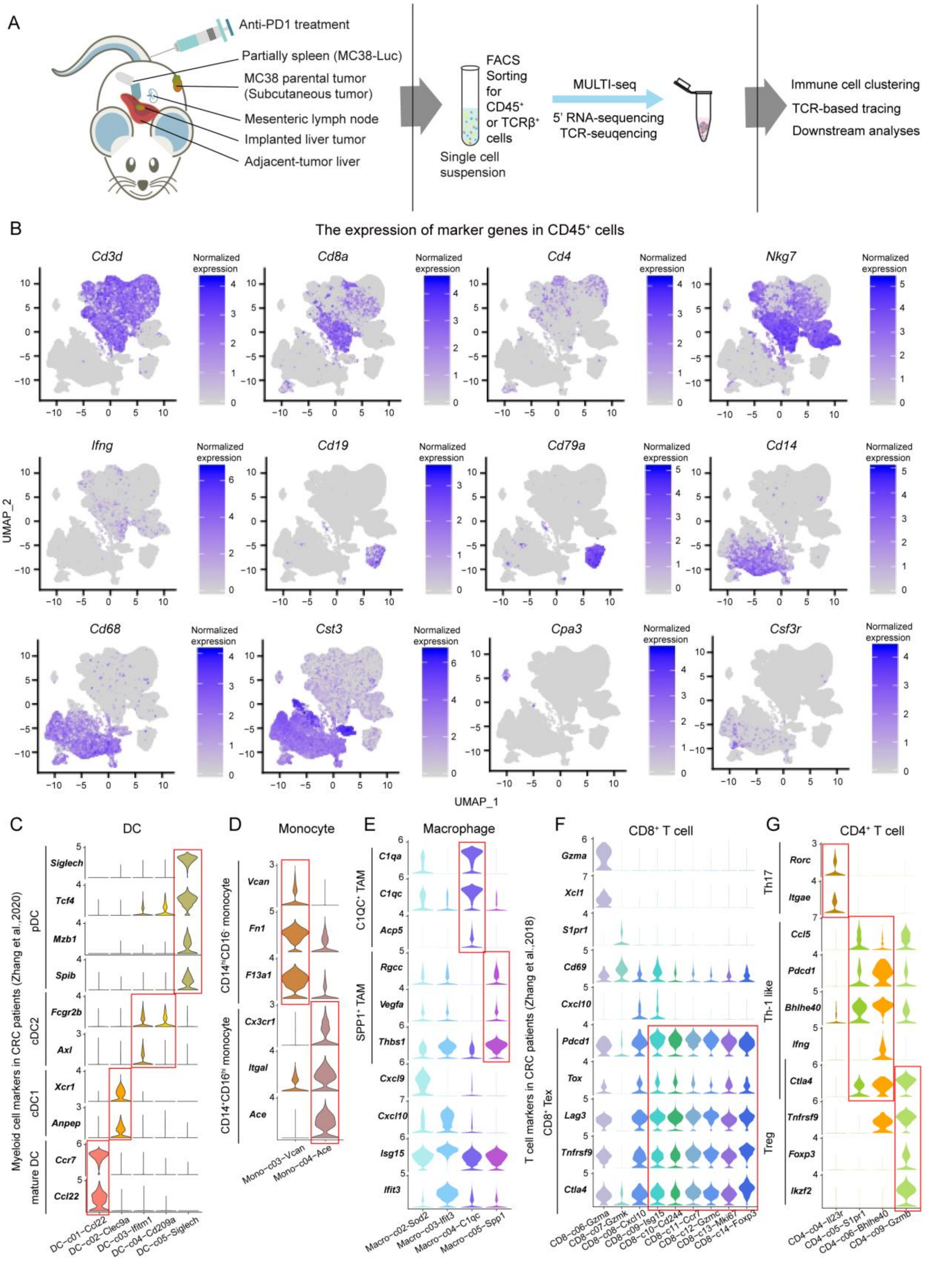

Figure S2. Characterization of the major cell types in CSLT mice based on scRNA-seq.

(A) Experimental design for scRNA-seq analysis of CSLT and SubQ-only mice.

(B) UMAP plots showing the expression of canonical marker genes for the major immune cell populations.

(C-G) violin plots showing the expression of marker genes based on CRC patients[14,16] in DCs (C), monocytes (D), macrophages (E), $CD8^+$ T (F) and $CD4^+$ T cells (G) clusters in mice. Colors represent different clusters.

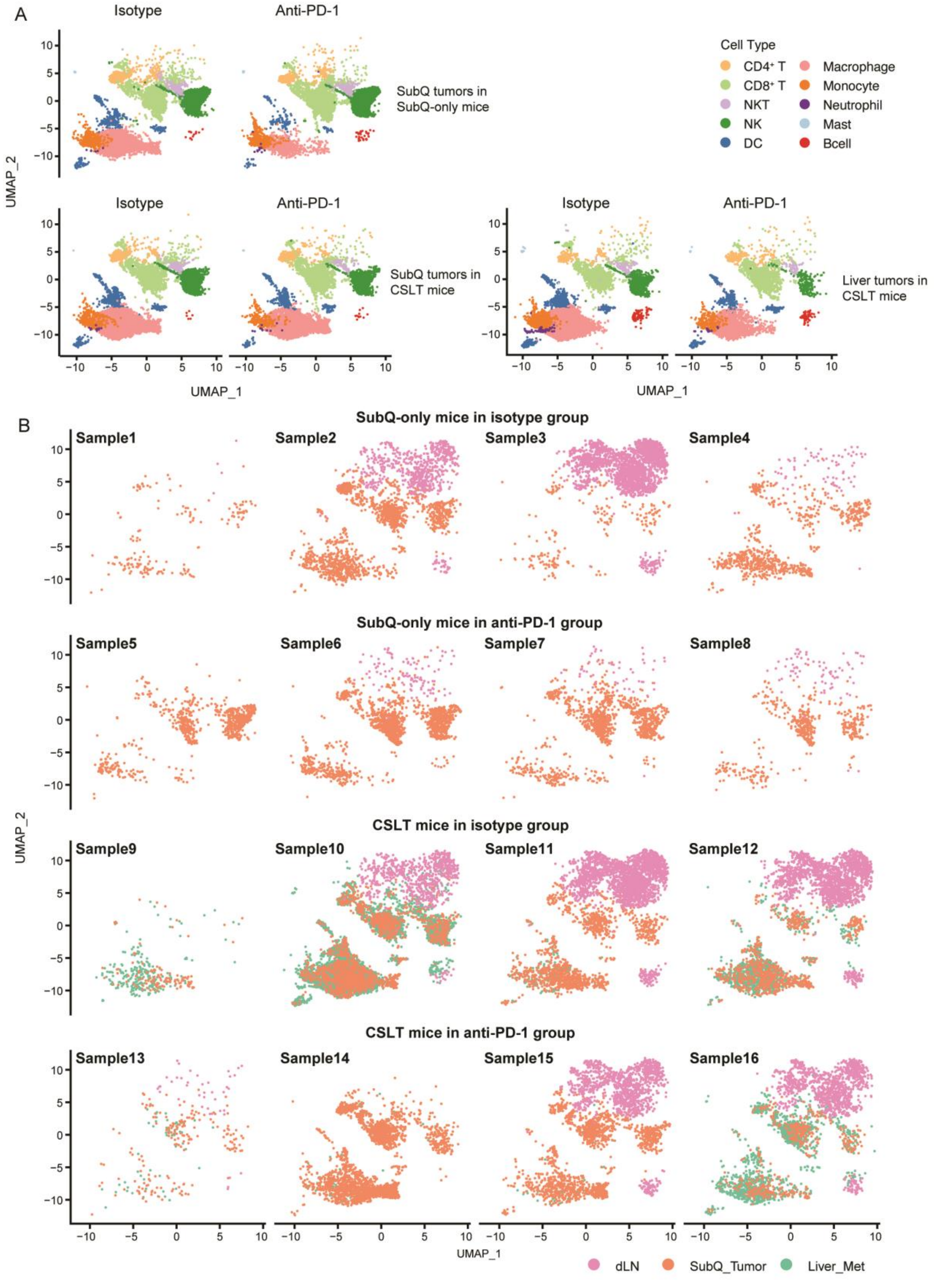

Figure S3. UMAP plots showing the cell distribution in different treatment groups of different mouse models.

(A) UMAP plots showing the cell type distribution in different treatment groups of different mouse models

(B) UMAP plot showing the cell distribution in individual mouse.

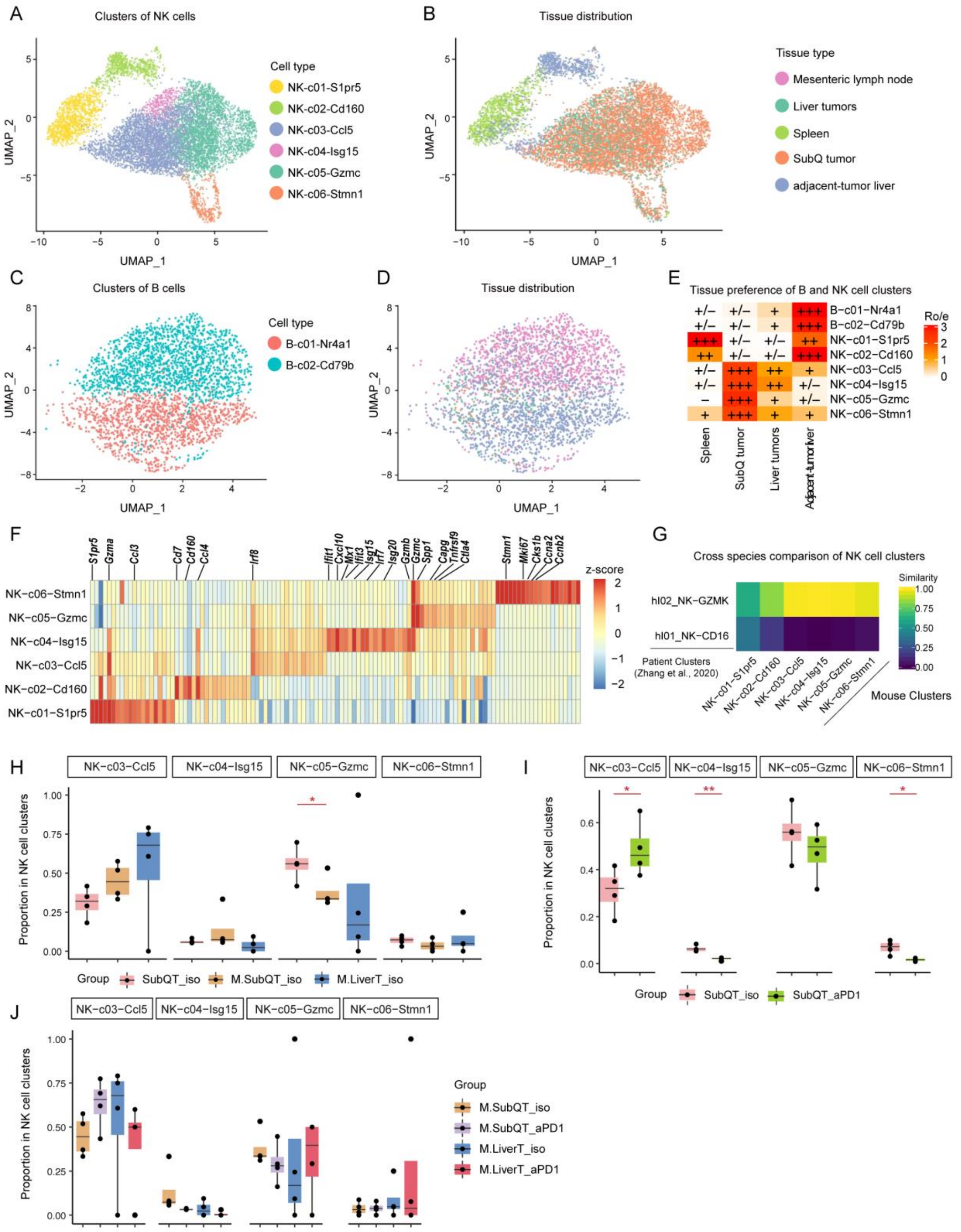

Figure S4. Characteristics and alterations of NK and B subsets in different treatment groups impacted by liver metastasis.

(A-D) UMAP plots showing the clusters of NK (A) and B (C) cells. Colors represent different clusters. UMAP plots showed the tissue distribution of NK (B) and B (D) cells. Colors represent different tissue types.

(E) Heat-map showing the tissue preferences of B and NK cells based on Ro/e.

(F) Heat-map showing the expression of marker genes of NK cell clusters.

(G) Heat-map showing the similarity of NK cell subsets in mice and in humans based on SciBet.

(H) boxplots showing the frequency changes of NK cell subsets in CSLT mice compared with SubQ-only mice. *, $p < 0.05$; **, $p < 0.01$; ***, $p < 0.001$. One-sided t test.

(I-J) boxplots showing the anti-PD-1-induced frequency changes of NK cell subsets in CSLT mice (J) compared with SubQ-only mice (I). *, $p < 0.05$; **, $p < 0.01$; ***, $p < 0.001$. One-sided t test. SubQT: subcutaneous tumors in SubQ-only mice; M.SubQT: subcutaneous tumors in CSLT mice; M.LiverT: liver tumors in CSLT mice; iso: isotype; aPD1: anti-PD-1 treatment.

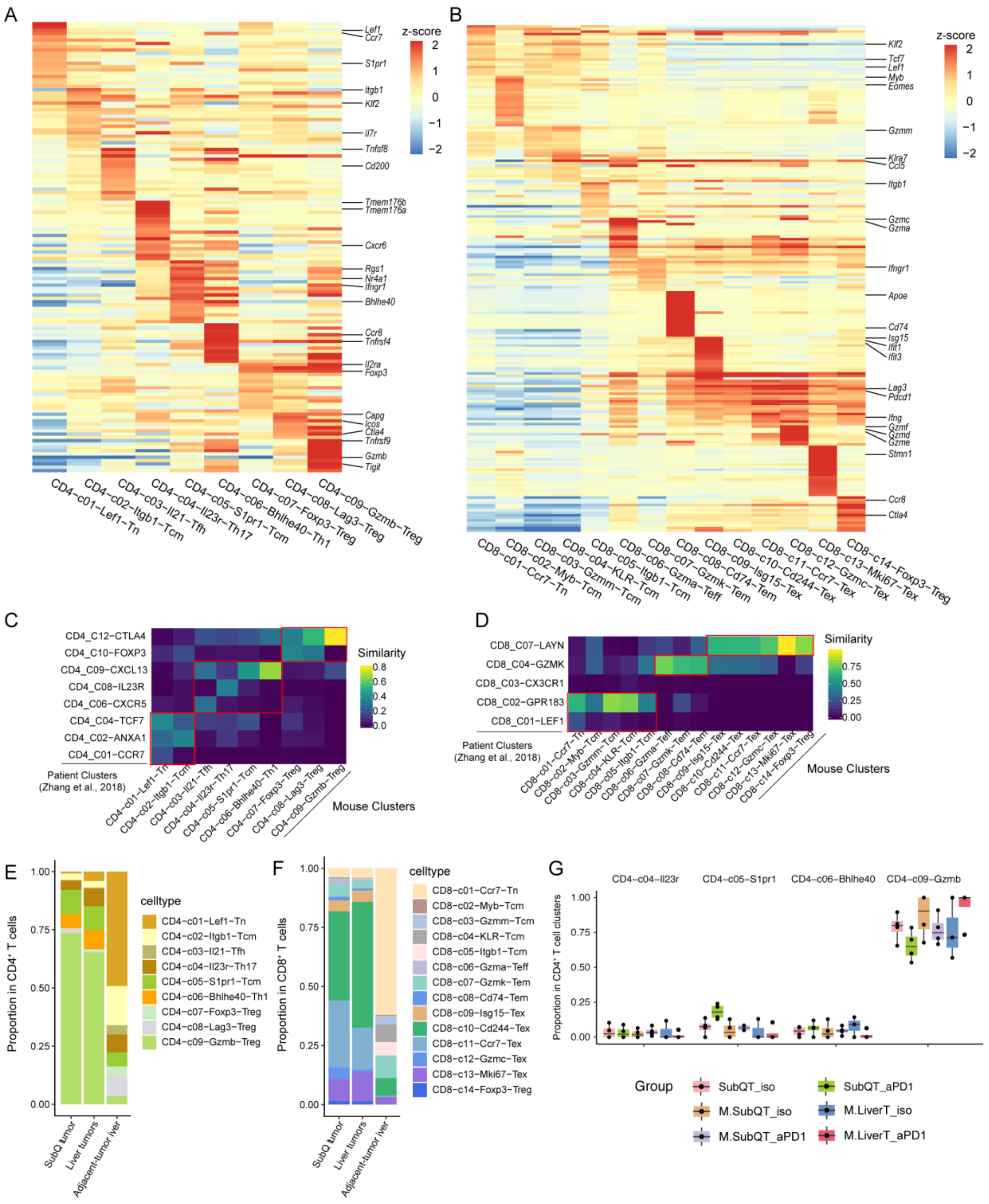

Figure S5. Characteristics of CD4^{+} T and CD8^{+} T cell clusters.

(A-B) Heat-map showing the expression of marker genes for CD4^{+} T (A) and CD8^{+} T cell subsets (B).

(C-D) Heat-map showing the similarity of $CD4^+$ T (C) and $CD8^+$ T cell subsets (D) in mice and in humans based on SciBet.

(E) Bar plot showing the proportion of $CD4^+$ T cell subsets in different tissues

(F) Bar plot showing the proportion of $CD8^+$ T cell subsets in different tissues.

(G) Boxplots showing that anti-PD-1 barely impact frequencies of $CD4^+$ T cell clusters. One-sided t test. SubQT: subcutaneous tumors in SubQ-only mice; M.SubQT: subcutaneous tumors in CSLT mice; M.LiverT: liver tumors in CSLT mice; iso: isotype; aPD1: anti-PD-1 treatment.

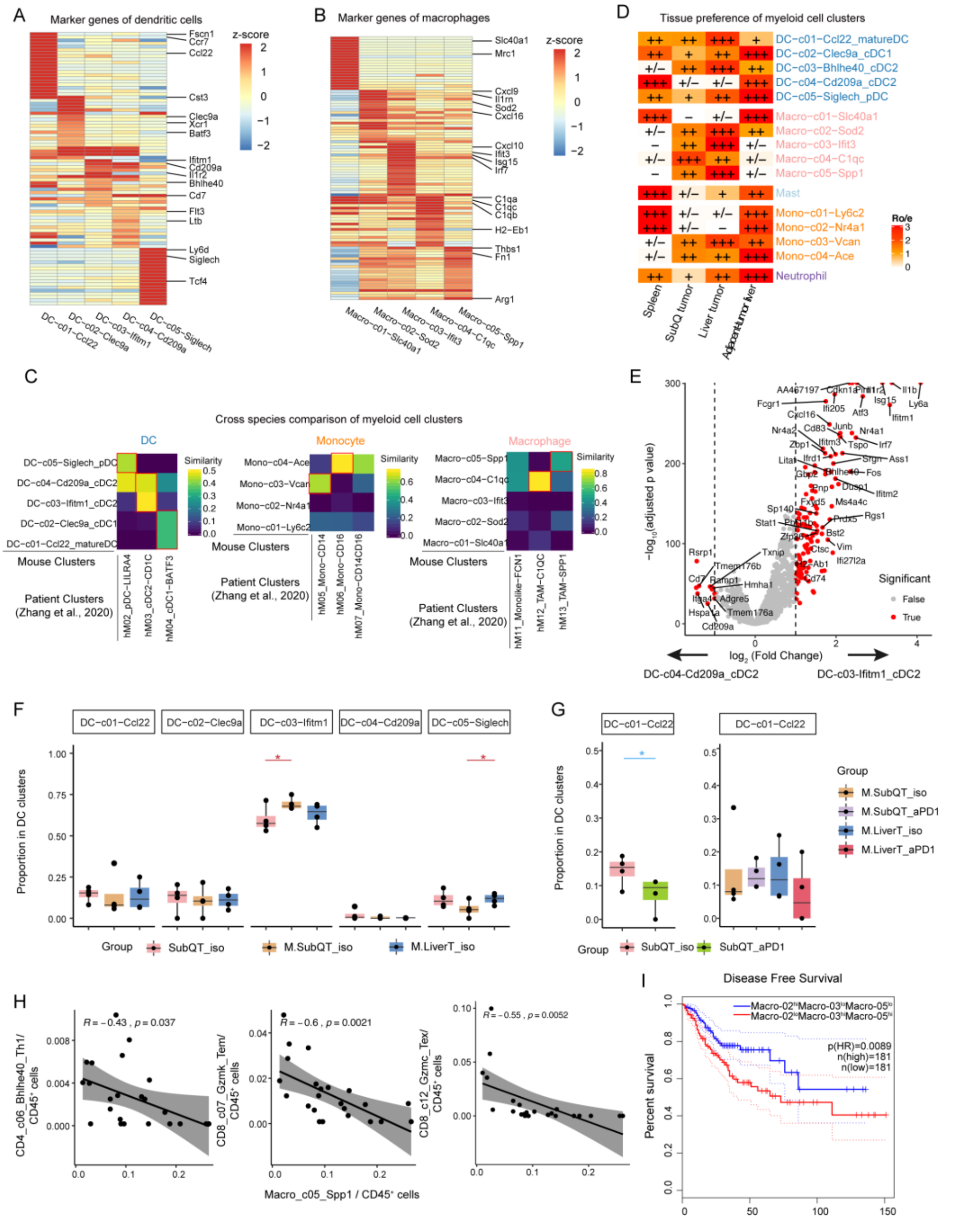

Figure S6. Characteristics of myeloid subsets following the anti-PD-1 treatment in CSLT and SubQ-only mice.

(A-B) Heat-map showing the expression of marker genes for DC (A) and macrophage subsets (B).

(C) Heat-map showing the similarity of myeloid subsets in mice and in humans based on SciBet.

(D) Heat-map showing the tissue preferences of myeloid subsets based on Ro/e.

(E) Volcano plot showing the differentially expressed genes of the two cDC2 subsets.

(F) Boxplots showing the frequency changes of DC subsets in CRLM mice compared with non-metastatic mice. One-sided t test.

(G) Boxplots showing the anti-PD-1-induced reduction of DC-c01-Ccl22 in CRLM mice compared with non-metastatic mice. One-sided t test.

(H) Scatter plots showing the frequency correlation of Macro-05-Spp1 with T cell subsets.

(I) Kaplan-Meier disease-free survival (DFS) curves of TCGA COAD and READ patients grouped by the gene signature expression of Macro-02-Sod1, Macro-03-Ifit3 and Macro-05-Spp1. HR, hazard ratio. Multivariate Cox regression.

SubQT: subcutaneous tumors in SubQ-only mice; M.SubQT: subcutaneous tumors in CSLT mice; M.LiverT: liver tumors in CSLT mice; iso: isotype; aPD1: anti-PD-1 treatment.

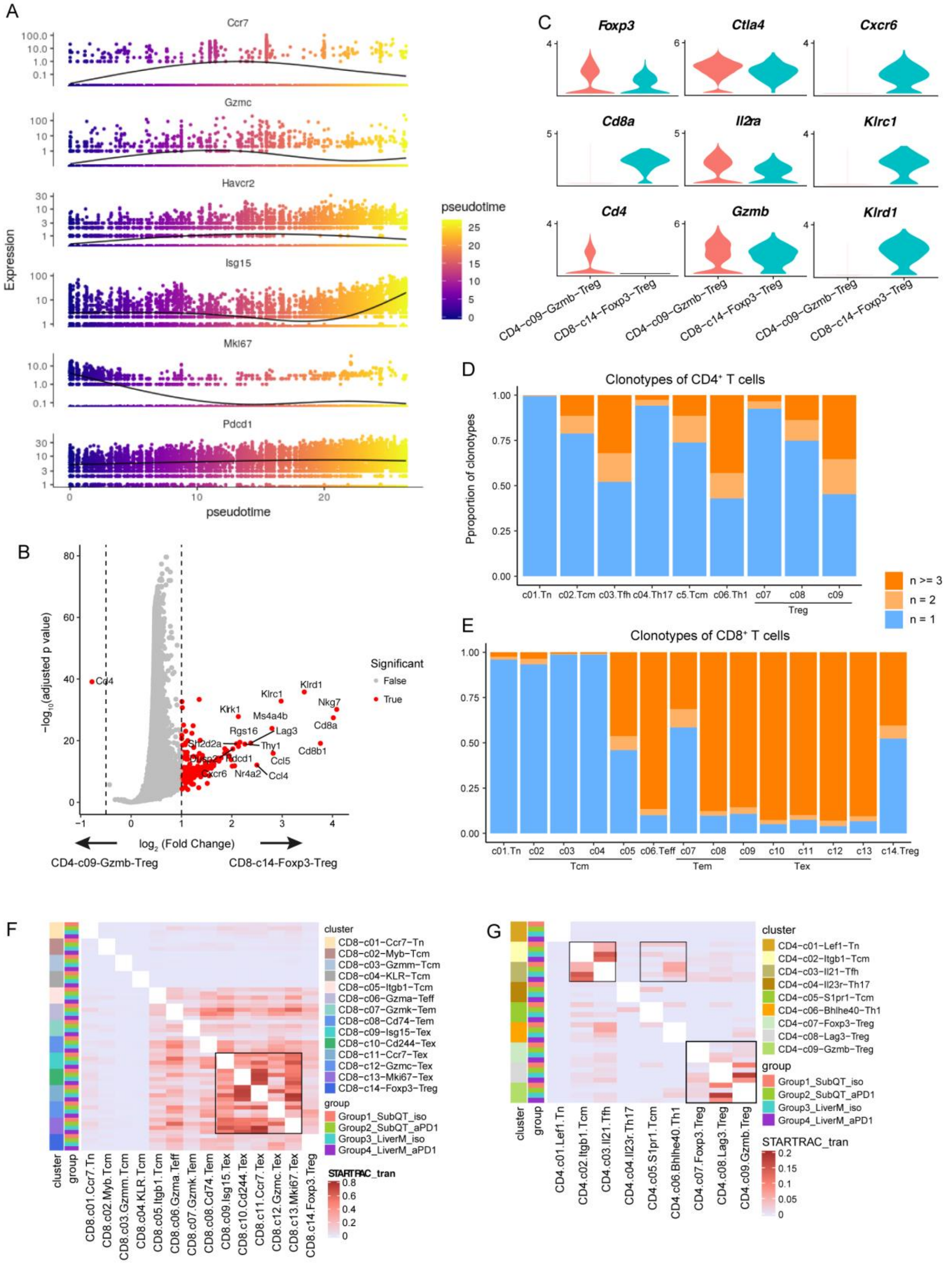

Figure S7. Expression features, clonotype information, and TCR sharing analyses of T cell subsets.

(A) Dot plots showing specific gene expression of Tex subsets along pseudotime.

(B) Volcano plot showing the differentially expressed genes between Cd8-c14-Foxp3 and Cd4-c09-Gzmb.

(C) Violin plots showing specific gene expression in Cd8-c14-Foxp3 and Cd4-c09-Gzmb.

(D-E) Bar plots showing the fractions of clonal cells in $CD4^+$ T (D) and $CD8^+$ T cell subsets (E).

(F) Heat-map showing the pairwise STARTRAC-tran indices of $CD8^+$ T cell subsets.

(G) Heat-map showing the pairwise STARTRAC-tran indices of $CD4^+$ T cell subsets.

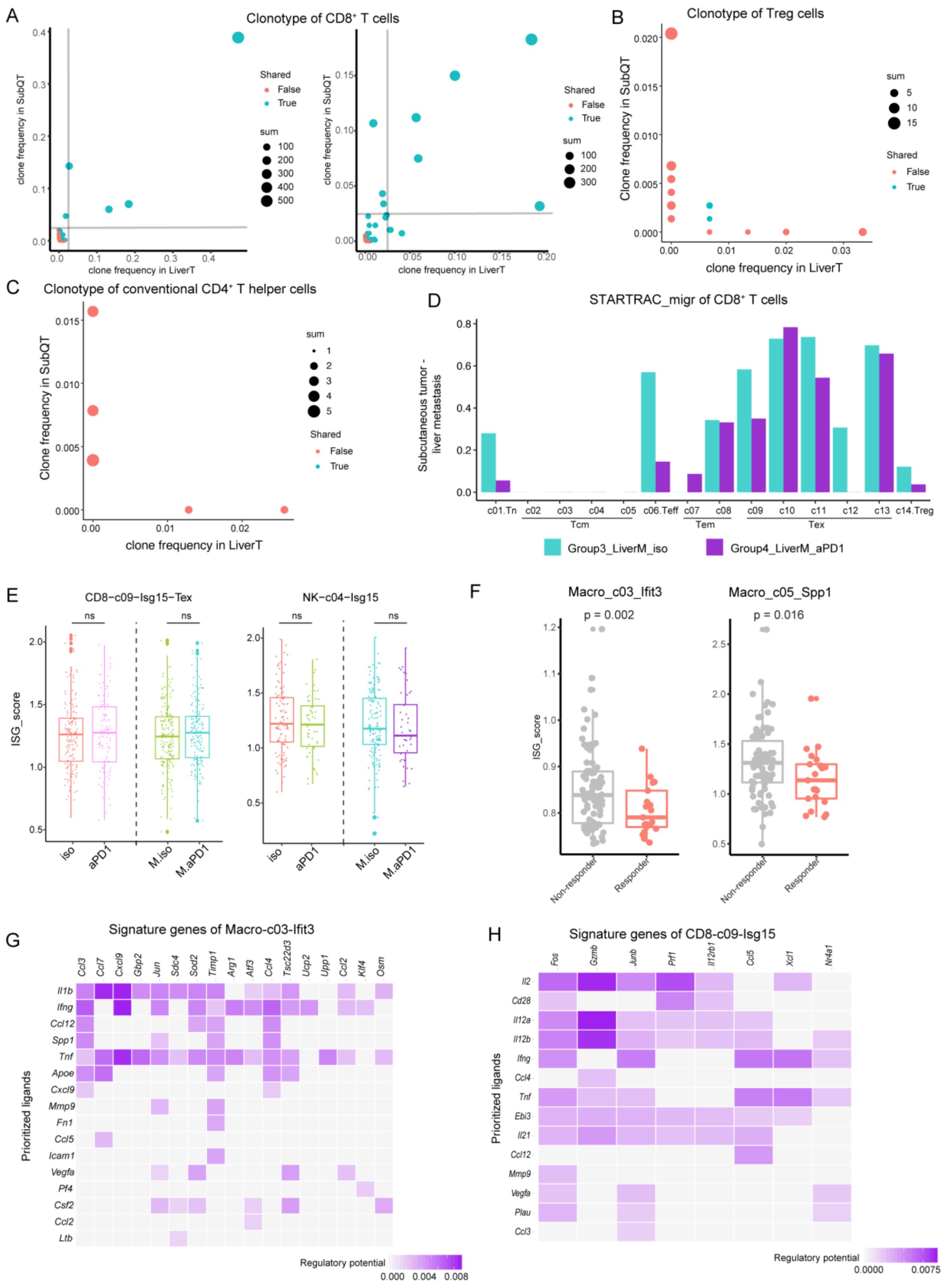

Figure S8. TCR sharing analyses of T cell subsets and NicheNet analysis of ISG-expressing populations.

(A) Scatter plots showing the shared clonotypes in CD8$^+$ T cells between SubQ and liver tumors, divided by treatment groups.

(B-C) Scatter plots showing the shared clonotypes in Tregs (B) and conventional CD4$^+$ T cells (C) between SubQ and liver tumors.

(D) Bar plots showing the pairwise STARTRAC-migr between SubQ and liver tumors of CD8$^+$ T cell subsets.

(E) Boxplots showing the ISG score changes of CD8-09-Isg15 and NK-04-Isg15 in different treatment groups. One-sided t test.

(F) Boxplots showing the ISG scores of different cell macrophage clusters in responders and non-responders of melanoma patients treated with anti-PD-1.

(G-H) Heatmap showing potential ligands driving the activation of Macro-c03-Ifit3 (G) and CD8-c09-Isg15 (H).

Supplementary Table 1. Cell number of each cell cluster.

Supplementary Table 2. Marker genes of B cell clusters.

Supplementary Table 3. Marker genes of NK cell clusters.

Supplementary Table 4. Marker genes of T cell clusters.

Supplementary Table 5. Marker genes of Myeloid cell clusters.

Supplementary Table 6. Clonotype scores of T cells in SubQT in different groups.
